# Modelling a multiplex brain network by local transfer entropy

**DOI:** 10.1038/s41598-021-93190-z

**Published:** 2021-07-30

**Authors:** Fabrizio Parente, Alfredo Colosimo

**Affiliations:** Department of Anatomy, Histology, Forensic Medicine and Orthopedics, Sapienza, University of Rome, Via Borelli, 50 00100 Rome, Italy

**Keywords:** Network topology, Computational neuroscience, Neurophysiology

## Abstract

This paper deals with the information transfer mechanisms underlying causal relations between brain regions under resting condition. fMRI images of a large set of healthy individuals from the 1000 Functional Connectomes Beijing Zang dataset have been considered and the causal information transfer among brain regions studied using Transfer Entropy concepts. Thus, we explored the influence of a set of states in two given regions at time t (A_t_ B_t_.) over the state of one of them at a following time step (B_t+1_) and could observe a series of time-dependent events corresponding to four kinds of interactions, or causal rules, pointing to (de)activation and turn off mechanisms and sharing some features with positive and negative functional connectivity. The functional architecture emerging from such rules was modelled by a directional multilayer network based upon four interaction matrices and a set of indexes describing the effects of the network structure in several dynamical processes. The statistical significance of the models produced by our approach was checked within the used database of homogeneous subjects and predicts a successful extension, in due course, to detect differences among clinical conditions and cognitive states.

## Introduction

Recently developed multivariate statistical methods allow to model brain fMRI signals in a large-scale network where brain regions can be associated based on their functional behaviour. The classical measure used in such a context is the functional connectivity, defined as the statistical dependence, usually calculated by temporal correlation, between spatially remote regions^1^. The above approach, however, has some weak sides since: (1) it is a symmetric measure and the influences among regions appear biunivocal, which impairs to explore causal relations; (2) it assumes a steady relationship between brain regions, while recent evidence^2,3^ show that a time-varying measure provides a more detailed information. Dealing with the first point, Friston^1^ introduced the Effective Connectivity (EC) proposing the Dynamic Causal Modelling (DCM) as a tool to extrapolate causal interactions^4^. Ramsey et al.^5^, carefully listed a number of problems that can affect modelling effective connectivity representative of causal relations by means of directed graphs—where nodes are brain regions—including the influence of unmeasured and latent (as well as random) variables. Those authors suggest machine learning techniques based upon feed forward acyclic algorithms^6^ for addressing the above problems. Other methods refer to Granger Causal Analysis^7^ and Dynamical Graphics^8^. Concerning the second point, several approaches were proposed, among which the time-windows correlation^2^ and the Co-Activation Patterns (CAP) analysis^3^. Some recent works give a more complete picture of brain dynamics combining the causal analysis and the time-varying with a detailed characterization of different states in different subjects^9^.

Another relevant issue in the study of interacting brain regions is the assumption that functional connectivity values can be split into positive and negative^10–13^, corresponding to in-phase and anti-phase signal dependence, respectively. As for the negative functional connectivity, although the pre-processing methods (e.g., Global Signal Regression, GSR) could insert spurious anti-correlation in fMRI data^14^, significant anti-phase signal dependences were found by different methodologies: intracranial electrophysiological measurements^11,15^, magnetoencephalographic (MEG) recordings^16^, as well as DCM^17^. Moreover, within the fMRI functional connectivity analysis, changes of anti-correlations have been related to some phenotypic and clinical features as working memory^18^, aging^18^, schizophrenia^19^ and NMDA receptors^10,20^.

In the present paper we report on the information transfer mechanisms proposing a new estimate of causal relations by Transfer Entropy (TE) values between brain regions from fMRI data under resting condition. TE allows to reckon the influence of the state of two agents (nodes) at a given time over the state of one of them at a following time^21^. Since the reciprocal influence between the two agents is not symmetrical a directional relation can be estimated. In addition, taking advantage of the methods described by Lizier et al*.*^22^, different kinds of influence (or transfer mechanism) between brain regions could be singled out and evaluated by a data-driven statistical analysis. It should be noticed that different information transfer processes can influence one another so that the ongoing brain activity cannot be estimated just by summing up, as if they were independent, the effects of each process.

The multiplex network analysis provides a useful tool to explore many complex features of networks^23,24^ by modelling different kinds of relationships between nodes as different single-layer networks. In other words, a multiplex network is generated by several kinds of interactions among the same set of nodes (e.g. people using different social media at the same time) and was successfully used in several contexts like diffusion processes^25^, information and epidemic spreading^26^, etc.. Thus, we decided to use a set of indexes to extract dynamical information on the whole functional brain network, in the frame of a multiplex (multi-layer) description. In summary, we propose to estimate and characterize functional causal interactions among brain regions on the basis of the TE and Multiplex Network concepts. A detailed account of the data pre-processing and TE values reckoning is provided in ‘Statistical characterization of interaction rules’ section; the interaction rules among brain regions are characterized in ‘Classification of interaction rules’ section; and the multiplex network approach in ‘The multiplex network’ section. For a synthetic overview of the paper workflow see Suppl. Mat. 1.

## Materials and methods

### Data collection and pre-processing

The 1000 Functional Connectomes Classic collection, Beijing Zang dataset, was used (https://fcon_1000.projects.nitrc.org/indi/retro/BeijingEnhanced.html). The database includes 180 brain functional images of healthy individuals acquired in a resting-state condition at the Beijing Normal University in China, the study was approved by the Institutional Review Board of Beijing Normal University Imaging Center for Brain Research. The functional images were acquired with a 3.0 T Siemens scanner and the following characteristics: 240 EPI volumes; repetition time, 2000 ms; echo time, 30 ms; slices, 33; thickness, 3 mm; gap, 0.6 mm; field of view, 200 × 200 2 mm; resolution, 64 × 64; flip angle, 90. The corresponding anatomical images, including a T1-weighted sagittal three-dimensional magnetization prepared rapid gradient echo (MPRAGE) sequence, was acquired covering the entire brain: 128 slices, TR (repetition time) = 2530 ms, TE (echo time) = 3.39 ms, slice thickness = 1.33 mm, flip angle = 7, inversion time = 1100 ms, FOV = 256 × 256 mm, and in-plane resolution = 256 × 192. The pre-processing step was performed as follows: the functional images were oriented to the twentieth scan, realigned and co-registered to the T1 image. Both the functional and the anatomical images were normalized to standard space (EPI image in Montreal Neurological Institute coordinates) using the normalization parameters of the T1 image. Then, a spatial gaussian filter was used (4 × 4x4 mm), the motion parameters were regressed out and a band-pass filtering in the range 0.008–0.09 Hz used. Afterward, the images were corrected by the anatomical CompCorr method^27^. SPM8 (Statistical Parametric Mapping, Wellcome Department of Cognitive Neurology, London, UK) and the Functional Connectivity Toolbox (CONN) were used in the previous steps, on a MATLAB R2010b platform.

The images of each subject were divided into 90 ROIs by the automatic anatomical labeling^28^ and from each ROI the time series were extracted. The short names used to identify the ROIs throughout this paper are listed in the Suppl. Mat. 2. The Framewise Displacement (FD) method^29^ was used to check the movement-linked variability in single scans. Movements larger than 0.2 mm were taken as an indication of bad scan and a temporal mask from the preceding to the following two scans (spanning 8 s) was also considered. Following Power et al*.*^29^ bad scans should be scrubbed off from the analysis. Although this operation does not affect the brain regions showing synchronous activation, however the dynamic among regions is influenced, and we decided to remove the subject if bad scans > 30 (lasting more than 60 s). As a result, 23 subjects were filtered out.

### Causal interactions estimate

**Figure 1:**
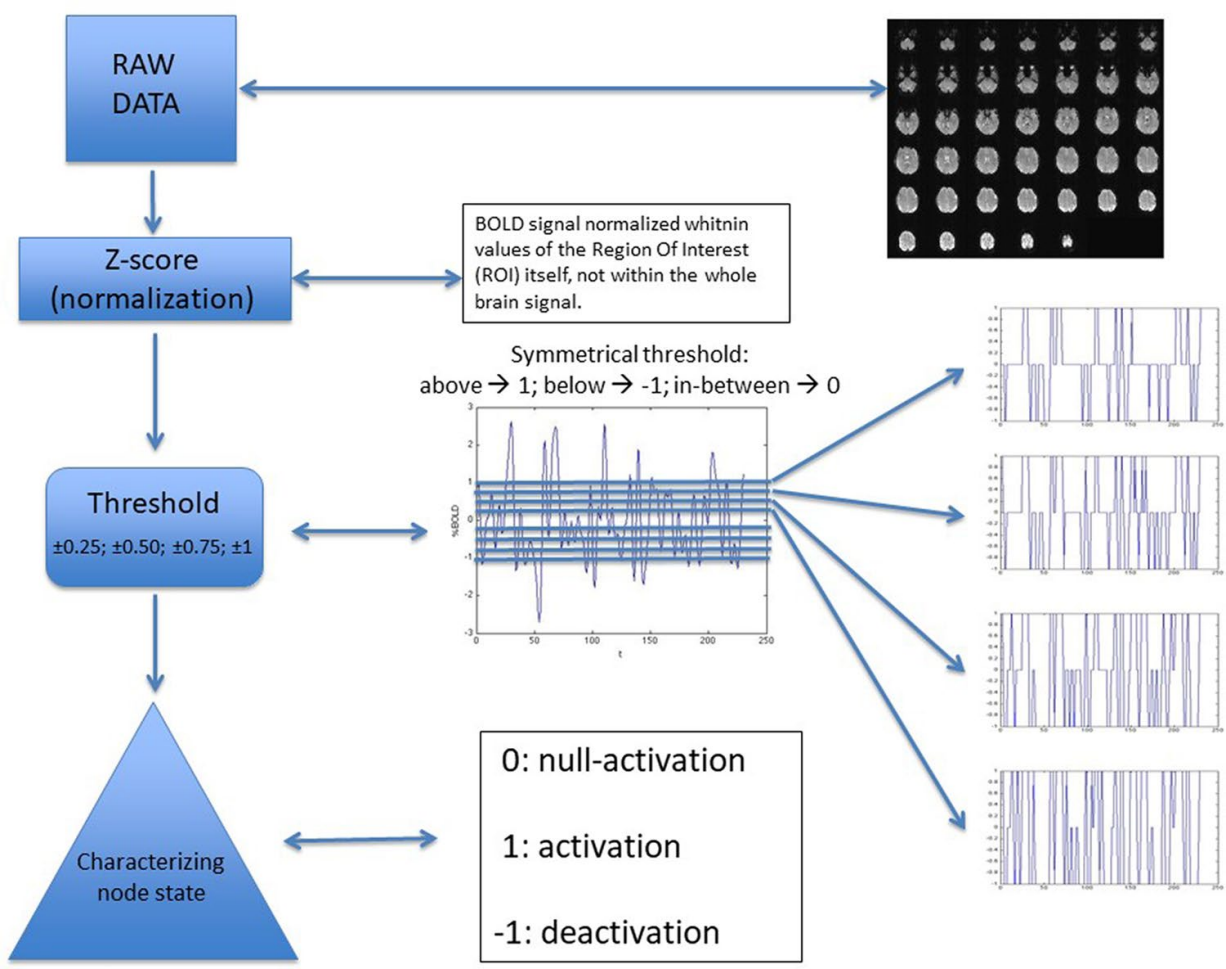
Flow chart of brain signal processing. Brain signals of each subject were submitted, after pre-processing, to the following procedure: Z-score normalization of time-series; thresholding by four symmetrical thresholds; discretization of signal values in: 1, − 1 and 0. Thus, each signal is redefined as a dynamic process involving: activation (1), de-activation (− 1) and null-activation (0).

In order to pick up significant events, we filtered the z-score normalized time-series by four increasing thresholds of standard deviation fractions^30,31^: (+ /-)0.25; (+ /-)0.50; (+ /-)0.75; (+ /-)1. In such a way, the signal complexity was reduced to single events in which (because of the symmetric threshold) three possibilities are considered: 1; − 1; 0, whether the signal is above the positive threshold, below the negative threshold, or in between the positive and negative thresholds, respectively (see Fig. 1). Moreover, the three above conditions point to possibly significant associated events: activation (1); deactivation (− 1); null-activation (0). Notice that beside the state of null-activation, not only activation but also deactivation are possibly significant events. Thus, to account for the anti-correlated interactions^10–13^, we distinguish the null-activation (0) from the deactivation (− 1).

To elucidate time-dependent interactions, we need the state of a node (B) at a given time step (n), and at the following step (n + 1). We also need the state of another node (A) at the time step n, likely interacting with the state of (B): A_n_(state) + B_n_(state) → B_n+1_(state), where the arrow indicates the time flow and (state) defines the activity condition (1, − 1, 0) of the node. Given the 3 possible conditions (0, 1 and − 1) and the 3-time dependent states (A_n_, B_n_ and B_n+1_), 3^3^ = 27 possible combinations describe the interaction among the two nodes. Hereafter, we call "rules" such combinations of interactions.

**Figure 2:**
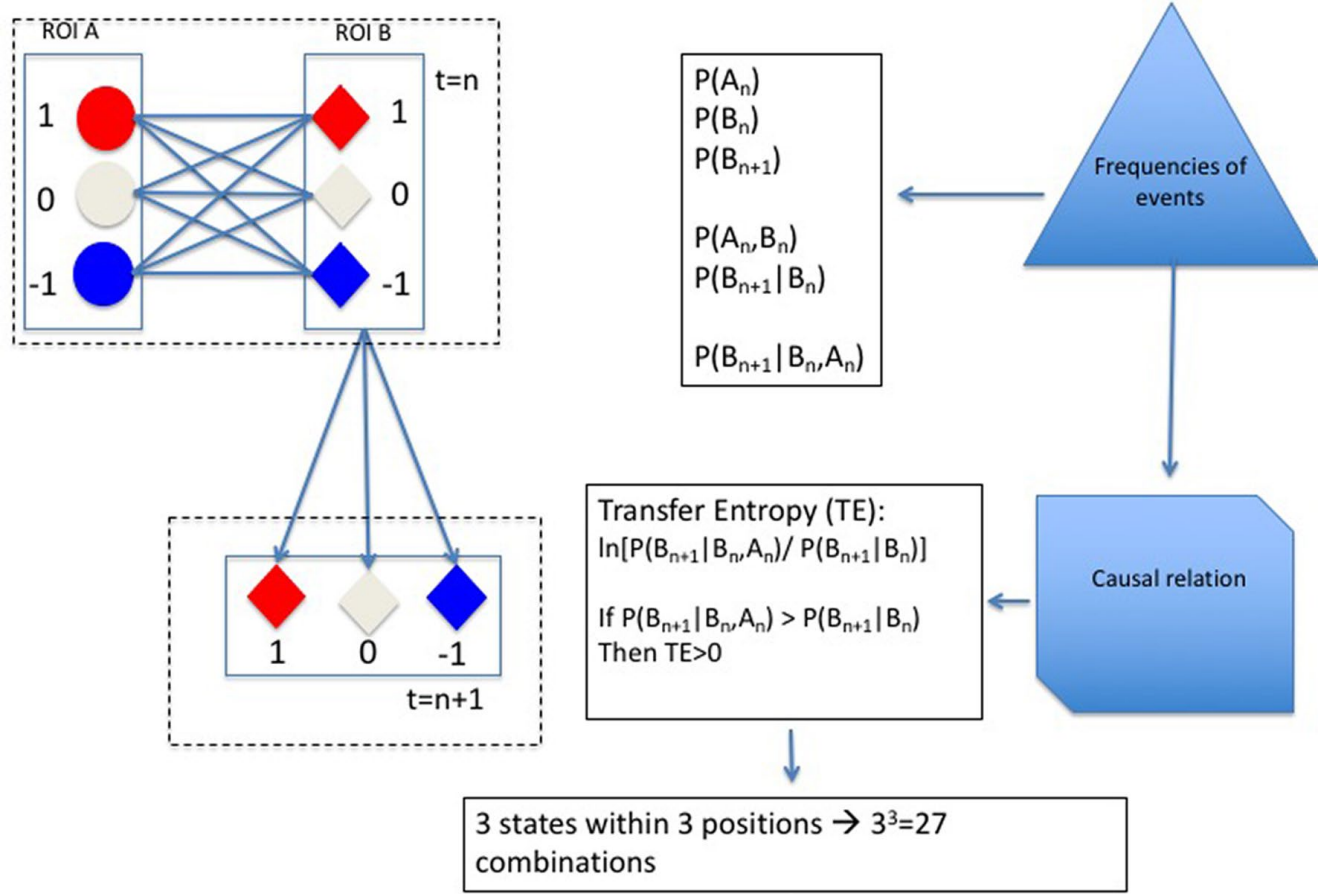
Characterization of dyadic interactions. Given the state value of two ROIs (**A**, **B**) at the time step (n) and the state of one of them (**B**) at the following time step (n + 1), a causal relation from (**A**) to (**B**) can be estimated by the Transfer Entropy (TE) through the calculation of: P(A_n_), marginal frequency of A in a particular state (1, − 1 or 0) at the time step n; P(B_n_) and P(B_n+1_), marginal frequencies of B in a given state (1, − 1 or 0) at the time step n and at the time step n + 1; P(A_n_;B_n_), joint frequency of A and B in a particular state at the time step n; P(B_n+1_|B_n_), conditional frequency of B in a particular state at the time step t = n + 1 taking into account the marginal frequency P(B_n_); P(B_n+1_|B_n_;A_n_) , conditional frequency of B in a particular state at the time step t = n + 1 taking into account the joint probability P(A_n_;B_n_); $\mathrm{ln}\frac{P\left({B}_{n+1}|{A}_{n};{B}_{n}\right)}{P\left({B}_{n+1}|{B}_{n}\right)}$ : a direct comparison between the causal hypothesis P(B_n+1_|A_n_;B_n_) and the null-hypothesis P(B_n+1_|B_n_). Taking into account the three possible states (1, − 1 and 0) and the three positions (A_n_, B_n_ and B_n+1_) a total of 27 combination rules are possible.

The TE estimate described by the following equation ^21^ has been used to quantify the significant probability of each combination:

1 $$T_{{A~ \to ~B}} = \sum P (B_{{n + 1}} ;~A_{n}^{{\left( k \right)}} ;~B_{n}^{{\left( l \right)}} )\ln \frac{{P\left( {B_{{n + 1}} |A_{n}^{{\left( k \right)}} ;B_{n}^{{\left( l \right)}} } \right)}}{{P\left( {B_{{n + 1}} |B_{n}^{{\left( k \right)}} } \right)}}$$

where the state of nodes A and B is specified by: n = the current time step; n + 1 = the following time step; k and l = number of time steps before n related to the event P(A) and P(B), respectively. In our analysis the k and l parameters were set to 1. However, since the repetition time for the dataset is equal to 2 s, the time between two consecutive steps of the time series vector corresponds to 2 s. In Fig. 2 a concise scheme of the method is reported. The TE method deals with the difference (or Kullback–Leibler divergence^32^) between the interaction: $P\left({A}_{n+1}|{A}_{n}^{\left(k\right)};{B}_{n}^{\left(l\right)}\right)$; and the non-interaction hypotheses as described by a general Markov process: $P\left({A}_{n+1}|{A}_{n}^{\left(k\right)}\right)$. Thus, if the interaction hypothesis is verified, a positive value is assigned to that rule and all negative values are set to 0, assuming they indicate a misinformation transfer^33^. Among the 27 interactions also indicated as "rules", the not significant ones were filtered out by a series of statistical tests after reshuffling the original pre-processed BOLD signals for each subject. Thus, since only the time-sequences are randomized, the marginal frequency of events in each brain region is unchanged, and the local TE could be re-calculated for each brain region combination, for all subjects and rules (more details in the Suppl. Mat. 3). The correlation among significant rules was explored by a hierarchical clustering method^34^.

### Network analysis

The interaction rules can be represented by a series of not symmetrical networks, where links are not reciprocal (A → B ≠ A ← B). Accordingly, any interaction between nodes (ROIs) is described for each subject by a weighted and directional network (W = {w_i,j_}) where links are represented by weight (w) and direction. In such a frame, the nodes are characterized by the sum of the associated weights: ${s}_{i}={\sum}_{j}{w}_{i,j}$, where w_i,j_ is the weight of a link between nodes i and j. This quantity is called *node strength*. Moreover, due to a possible asymmetry between mutual interactions, *in-strength* and *out-strength* can be separately calculated by summing the weights of in- and out- connections (i.e., inverting subscripts i and j), and the total sum is defined *total-strength*. This can be also applied to directional binary networks (values = 1 or 0), producing in-, out-, and total- degrees.

#### Multiplex networks

In the case of a multiplex network the classical metrics of the network theory applies to each single-layer network included in the multiplex. Thus, for a global description, the introduction of new network indexes is required: a) in a system including N nodes and M layers, each layer α (with α = 1, …, M) is associated to an adjacency matrix ${A}^{\alpha }=\{{a}_{i,j}^{\alpha }\}$, where ${a}_{i,j}^{\alpha }$= 1 if nodes i and j are connected by a link in the α layer; b) in a weighted network (W) each ${a}_{i,j}^{\alpha }$ ≠ 0, is associated to a weight w; c) in a directional network links are from i to j. A multiplex network can be specified by the vector of adjacency matrices of the M layers as **W** = {W^[1]^, …,W^[M]^}, where ${W}^{\left[\alpha \right]}={\{w}_{i,j}^{\left[\alpha \right]}$} and we can define the strength of node i by the vector: **s**_**i**_ = {s_i_^[1]^, … , s_i_^[M]^} where i = 1, …,N, and the strength of node i in layer α as: ${s}_{i}^{\left[\alpha\right]}={\sum}_{j}{w}_{i,j}^{\left[\alpha \right]}$. As an alternative, a multiplex network is obtained by summing up all (or a set of) layers calculating the weighted aggregated overlapping adjacency matrix: ${O}^{w}=\{{o}_{i,j}^{w}\},$ where ${o}_{i,j}^{w}={\sum }_{\alpha }{w}_{i,j}^{\left[\alpha \right]}$. In this case the strength of node i, is calculated by the weighted overlapping degree: ${o}_{i}^{w}={\sum }_{j}{o}_{i,j}^{w}={\sum }_{\alpha }{s}_{i}^{\left[\alpha \right]}$. The directional version of this formalism is obtained by splitting the node strength into the corresponding in-, out- and total-strength. In summary, we introduce two ways to describe a multiplex network:

- the vector of adjacency matrices, **W**;
- the weighted aggregated overlapping adjacency matrix, **O**^**w**^.

The W contains all the information of the multiplex network preserving any difference between layers and allows to calculate any structure formed by the combination of all (or set of) layers. However, its combinatorial nature needs a huge computing time. The second approach, on the other hand, loses the architecture of the layers and, although faster, may miss the structural features of the network. To characterize the whole brain functional network we used both methods, comparing the results in each condition.

#### Network dynamics

The influence of the network structure on dynamical processes can be estimated by appropriate indexes in the framework of the analysis of reciprocity^35–37^ (RI) and of subgraphs^38,39^. The RI estimates the occurrence of mutual relationships among node pairs looking at the number of reciprocal links normalized to the total number of links^35^. In the case of weighted networks, however, the number of links (different for each connection type) cannot be easily calculated and, among the proposals introduced to face the problem, we chose the reciprocity method described in Squartini et al*.*^36^. An extension to a multiplex directional network^37^ introduced the notion of multireciprocity, i.e., the tendency of links in one layer to be reciprocated by links in another layer. In summary, RI values normalized to a null-model network in the range [+ 1, − 1] (see the Statistical Analysis Section below) indicate:

- reciprocate connections (RI > 0);
- reciprocation avoidance (RI < 0);
- independent behaviour (RI = 0).

**Figure 3:**
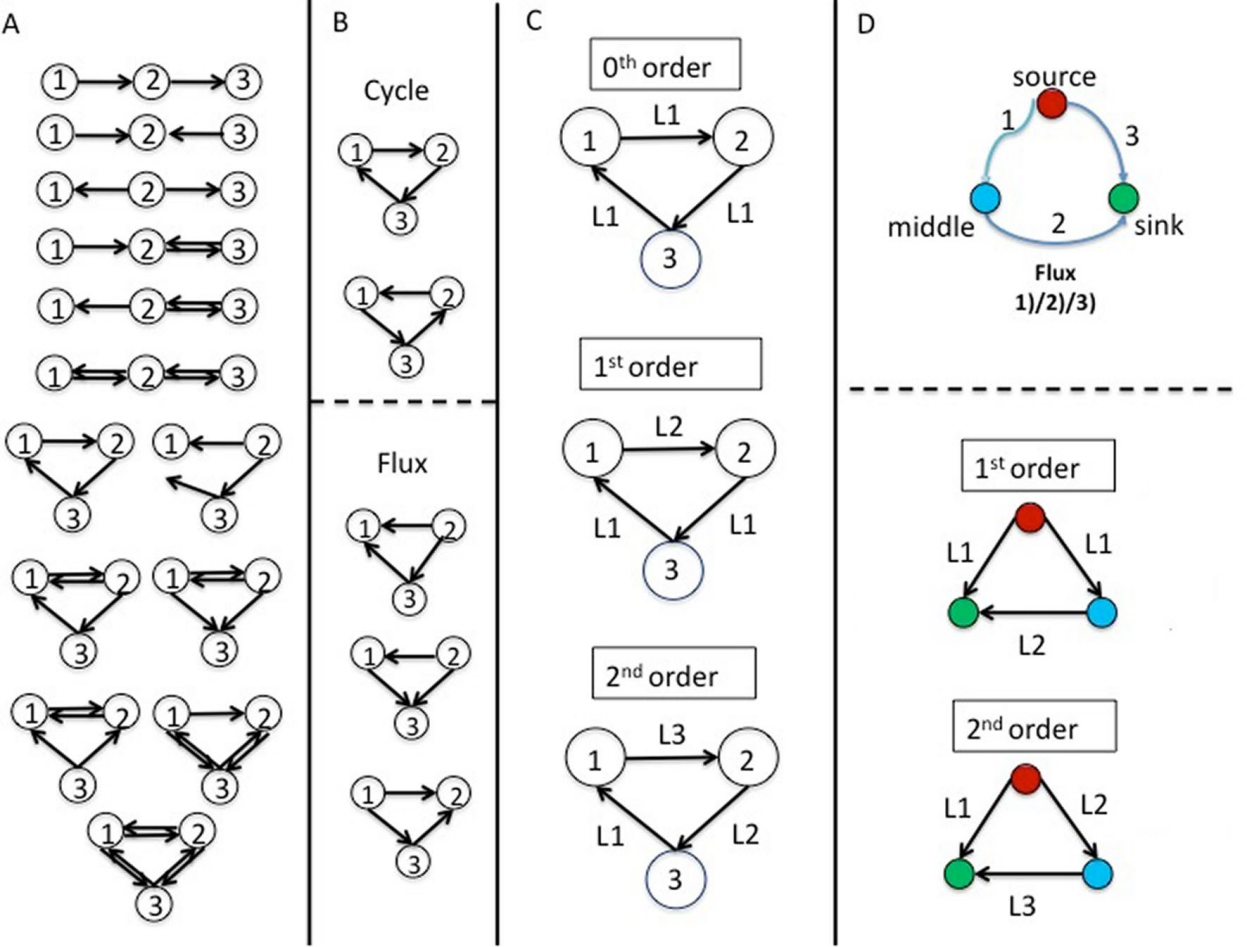
Triadic motifs and subgraphs. (**A**) the whole set of motifs within three nodes. (**B**) The Cycle (top) and Flux (bottom) subgraphs with the corresponding isophorms. (C) The three orders of Flux subgraphs in the multiplex network: 0^th^ order having all links in the same layer (M^1^); 1^st^ order with two links shared in a same layer (M^1^) and one in another layer (M^2^); 2^nd^ order in which each link belongs to different layers (M^1^, M^2^ and M^3^). (**D**) Functional characterization of nodes and links in a Flux subgraph (top); Models of Flux subgraphs of 1^st^ and 2^nd^ order used in this paper (bottom). For more details see the text and **Suppl. Mat. 4**.

Concerning subgraphs, the recent introduction of graphlets^38^, suggests the following definition of a subgraph: a combination of in- and out-links within a set of connected nodes where double links are not allowed. Notice that different combinations of in- and out-links can draw the same geometric pattern, called isomorphic, to be counted only once (see Fig. 3, panel A). According to Onnela^39^ we characterize a subgraph by two indexes: Intensity (I) and Coherence (C). The I index is defined as the geometric mean of the subgraph links:

2 $$I_{g} = \left( {\prod\limits_{{(i,j) \in l_{g} }} {w_{{i,j}} } } \right)^{{1/|l_{g} |}}$$

where g is a subgraph; w_ij_ is the weight of g between node i and j; l_g_ is the set of links in g and |l_g_| is their number. In such a way, only triads formed by non-zero links are taken into account (a link = 0 would nullify the product). Moreover, a continuum set of low intensity values in a subgraph indicates a possible noise effect of link weights. The C index is calculated as the ratio between the geometric and arithmetic average of subgraph weights and checks the similarity of weight values within a subgraph. Since high level of I could be due to a single link, C indicates whether a whole set of subgraph links participate to the I value. Note that we use C instead of Q (used by Onnela et al*.*^39^) in order to distinguish the Q index of modularity described below. The Intensity (I) and Coherence (C) indexes were used in this paper to quantify the magnitude as well as the statistical significance of each subgraph (see also Suppl. Mat. 4). In our work we studied the geometry of closed 3-nodes subgraphs in a directional network. Concerning the trajectories of subgraphs, we adopted the classification proposed by Takaguchi and Yoshida^40^, where two kinds of subgraphs are considered: Cycle and Flux triads (Fig. 3, panel B):

- Cycle triads is a subgraph of three connected nodes having non-zero weight in both in- and out-connections, namely a directional cycle;
- Flux triads is a connected subgraph in which one node has non-zero weight in both in- and out-connections, and the other two nodes only have in- and out- non-zero connections, respectively.

To characterize triads in a multiplex network we adopted the definitions reported by Battiston et al*.*^24^ (Fig. 3, panel C). A 0^th^ order triad is referenced by its type (Cycle or Flux) associated to the belonging layer. In the case of 1^st^ and 2^nd^ order Cycle subgraphs, the names are derived by the sequence of layers. Figure 3, panel D provides a schematic picture of the functional characterization of nodes and links.

#### Centrality and modularity analyses

In the Centrality analysis^41^ nodes can be sorted by specific features accounting for interactions within the whole system. The node degree is used to characterize node centrality and, in a directional and weighted network, the corresponding metrics. We defined as central the nodes having a strength value higher than, at least, the third quartile of their distribution and corresponding to the 75th percentile (upper quartile) of the dataset. In our case two kinds of central nodes are possible: in-strength nodes, where the cerebral regions tend to concentrate a major part of in-flux pathways, and out-strength nodes, where the brain regions are characterized by a major amount of out-flux pathway. Thus, in-strength and out-strength indicate nodes endowed with a major in-flux or out-flux, respectively; in the brain system central nodes could be the main sink in the first case and the main source areas in the second case.

In a multilayer description, besides the study of the node degree distribution in each layer, the distribution of central nodes across different layers was reckoned according to the following definitions:

- Local central node: if present in not more than 25% of the total layers;
- Intermediate multiplex central node: if present in the range between 25% and 75% of the total layers;
- Multiplex central node: if present in more than 75% of the total layers.

In binary and bidirectional networks, a widely used definition of Modularity^42,43^ accounts for nodes grouped into sets densely connected internally among each other through the Q index. In this way, a node can be included in a finite number of communities. The difference between the fraction of links in a community and the expected fraction of links in a null model is estimated as follows:

3 $$Q~ = ~\frac{1}{{2m}}~\mathop \sum \limits_{{ij}} \left[ {A_{{i,j}} ~ - ~\frac{{k_{i} ~*~k_{j} }}{{2m}}} \right]~\delta _{{ci,cj}}$$

where A_i,j_ is the link between nodes i and j in the adjacency matrix A; k_i_ and k_j_ are the node degrees of nodes i and j; m is the total number of links in the network and δ_ci,cj_ is the Kronecker delta symbol, where ci and cj are the labels of the community to which nodes i and j are assigned. In Eq. (3) the term A_i,j_ refers to the real network, and the term $\frac{{k}_{i}*{k}_{j}}{2m}$ is the probability of an edge between nodes i and j in a random graph. In a directional network a different approach is needed, and Newman updated the Q estimate to account for the direction of links^43^. Thus, the second term in Eq. (3) is defined as: $\frac{{k}_{i}^{in}*{k}_{j}^{out}}{m}$, where ${k}_{i}^{in}$ is the in- degree of node i, ${k}_{j}^{out}$ is the out- degree of node j, and m the total number of links. The random model for directional network refers to the probability distribution of nodes i and j being directly linked. In weighted networks the fraction of edges is substituted by the fraction of weights in a community. In Eq. (3) the matrix W_i,j_ is inserted as an adjacency weighted matrix and the $\frac{{s}_{i}^{in}*{s}_{j}^{out}}{{m}_{w}}$ as a null model, where ${s}_{i}^{in}$ and ${s}_{j}^{out}$ indicate the in- and out-strength on nodes i and j, respectively, and m_w_ is the sum of the whole set of weights in the network.

#### Reckoning network indexes

For the calculation of the previously described indexes an original script (available upon request from one of the authors, F.P.) was designed, except for the random models and the modularity analysis, where we used scripts from the MATLAB Brain Connectivity Toolbox^44^ (BCT). The global indexes were calculated for each subject separately, while the local (centrality) and the modularity analysis were carried out over mean matrices across all the subjects. For the randomized models, the algorithm proposed by Rubinov and Sporn^45^ were used, in which the weighted directional network is randomized preserving the in-strength distribution. However, since fully connected matrices are obtained from averaged networks, in the modularity analysis a combinatorial weight randomization was used. In all cases random models refer to averages of 100 randomizations.

### Ethical approval

All procedures performed in studies involving human participants were in accordance with the ethical standards of the institutional and/or national research committee and with the 1964 Helsinki declaration and its later amendments or comparable ethical standards.

### Informed consent

Informed consent was obtained from all individual participants included in the study.

## Results

### Statistical characterization of interaction rules

**Figure 4:**
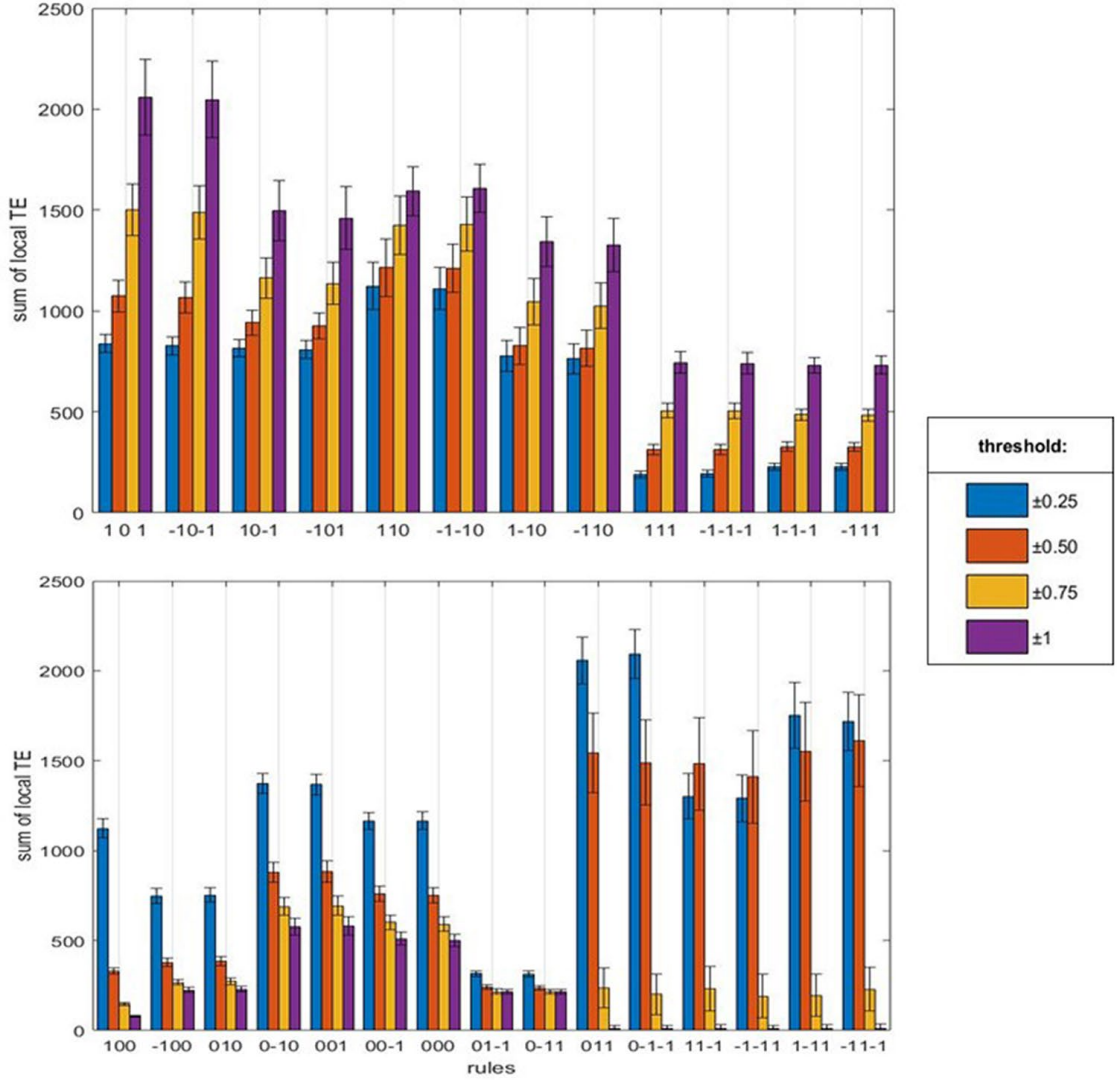
Sums of local TE in the interaction rules at increasing signal thresholds. The sums include the individual values of the subjects in the database (see the Methods). Top panel: rules with an increasing trend at increasing thresholds. Bottom panel: rules with mixed trends at increasing thresholds. Right panel: threshold values in the filtering procedure and colours association.

**Figure 5:**
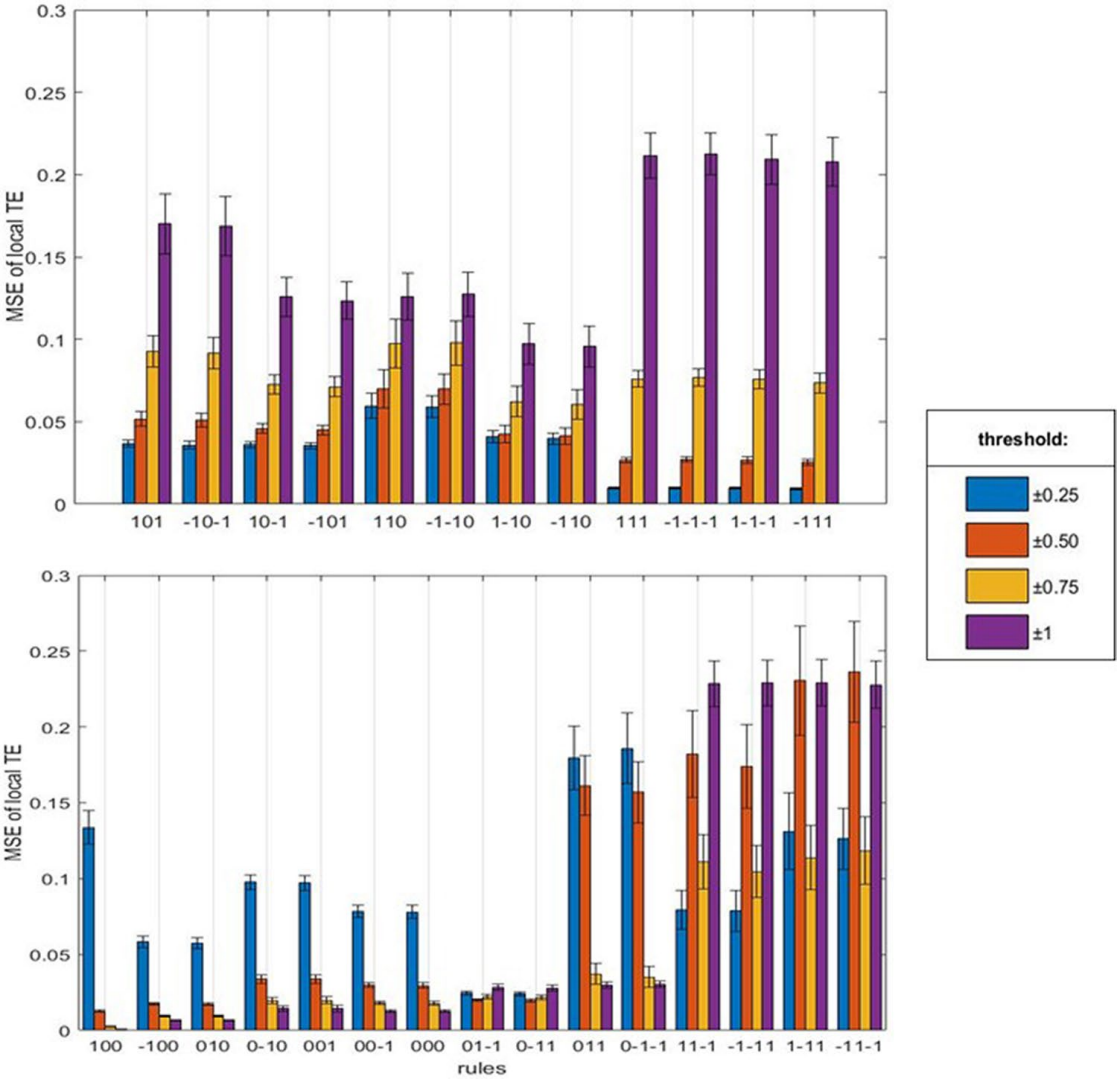
Mean Squared Error (MSE) of local TE as a function of signal thresholds. Top panel: rules with an increasing trend at increasing thresholds. Bottom panel: rules with mixed trend at increasing thresholds. For the meaning of columns and colours and the sequence of rules, see Fig. 4.

**Figure 6:**
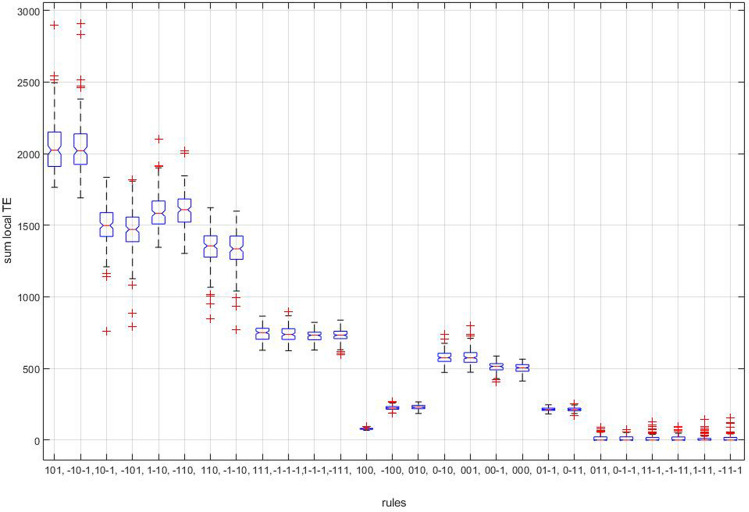
Post-hoc analysis of ANOVA one way. The distribution of local TE in the 27 different rules (see the text for details) shows that the first eight rules form a cluster independent from the others.

Different interaction rules show different patterns of TE values summed over all subjects as a function of the signal threshold: the rules showing an increasing and a decreasing trend are indicated in the top and bottom panels of Fig. 4, respectively. It is worth noting that the Mean Squared Error (MSE) analysis shows an increasing trend in the same twelve rules (Figs. 4 and 5, top panel) while the remaining rules show a decreasing, flat and fluctuating mixed behaviour (Fig. 5, bottom panel). A series of t-test for independent samples (right tail) were performed to compare real and random local TE at the highest signal threshold (1 S.D.) for the twelve rules in Fig. 4, top panel. The ANOVA analysis shows a significant effect of rules (*p* < 0.0001) at the higher signal threshold (1 S.D.) and in Fig. 6 the post-hoc analysis confirms a significant increasing of local TE for eight of the above mentioned twelve rules. Thus, eight rules share all the assumptions indicated in the statistical methods (see also Suppl. Mat. 3) and we restricted the analysis to those rules, where at least two of the previously described features, namely increasing trend of local TE and MSE at increasing threshold, appeared. Moreover, the result can be replicated by an unpaired t-student test (right-tail) performed for each subjects of the dataset independently for the following four rules (Table 1):

- A_n_ (1) + B_n_ (0) → B_n+1_ (1);
- A_n_ (− 1) + B_n_ (0) → B_n+1_ (− 1);
- A_n_ (1) + B_n_ (− 1) → B_n+1_ (0);
- A_n_ (− 1) + B_n_ (1) → B_n+1_ (0).

**Table 1:** Distribution of significant local TE among subjects.

Interaction rules	% of subjects
A_n_ (1) + B_n_ (0) → B_n+1_ (1)	100
A_n_ (− 1) + B_n_ (0) → B_n+1_ (− 1)	100
A_n_ (1) + B_n_ (− 1) → B_n+1_ (0)	100
A_n_ (− 1) + B_n_ (1) → B_n+1_ (0)	100
A_n_ (1) + B_n_ (0) → B_n+1_ (− 1)	97
A_n_ (− 1) + B_n_ (0) → B_n+1_ (1)	97
A_n_ (1) + B_n_ (1) → B_n+1_ (0)	83
A_n_ (− 1) + B_n_ (− 1) → B_n+1_ (0)	79
A_n_ (1) + B_n_ (1) → B_n+1_ (1)	0
A_n_ (− 1) + B_n_ (− 1) → B_n+1_ (− 1)	0
A_n_ (1) + B_n_ (− 1) → B_n+1_ (− 1)	0
A_n_ (− 1) + B_n_ (1) → B_n+1_ (1)	0

Fraction of 109 subjects with the same results for the different rules, *p* < 0.0001 Bonferroni corrected (157 repetitions).

The remaining four rules, namely:

- A_n_ (1) + B_n_ (0) → B_n+1_ (− 1);
- A_n_ (− 1) + B_n_ (0) → B_n+1_ (1);
- A_n_ (1) + B_n_ (1) → B_n+1_ (0);
- A_n_ (− 1) + B_n_ (− 1) → B_n+1_ (0);

showed results with a higher variability between subjects. To remove any potential source of noise we excluded from any further analyses 49 subjects (corresponding to the 31% of the database) since they did not produce significant (t-test independent sample) results in the distribution study reported in Table 1. A final relevant observation concerns the following rules:

- A_n_ (1) + B_n_ (1) → B_n+1_ (1);
- A_n_ (− 1) + B_n_ (− 1) → B_n+1_ (− 1);
- A_n_ (1) + B_n_ (− 1) → B_n+1_ (− 1);
- A_n_ (− 1) + B_n_ (1) → B_n+1_ (1);

characterized by an increasing trend in the MSE, and not significant results in the right-tailed (although significant in the left tailed) comparison test (not shown). Thus, the decrease of their local TE at increasing thresholds indicates a lower probability of expression in the time vector than in the random counterpart. Such rules can be reduced to two couples: in the first couple the target node (B) assumes the same state of the previous time step if the source node (A) falls in the same state; in the second couple, the target node (B) assumes the same state of the previous time step if the source node (A) falls in the opposite state. Thus, both couples can represent a state preservation process, since they maintain their state in time given an equal or opposite state of the source node (A). We may argue that such combinations are less favoured than others and that an underlying mechanism stands in the way of this dynamic. It seems that brain regions can be characterized by single and unique events, in agreement with previous findings^29,46^. However, it must be considered that only few peaks survive at the highest threshold level, so that a persistent value of the same state over time could anyway be less likely. In addition, an effect due to the reshuffling/randomizing method cannot be excluded.

### Classification of interaction rules

**Figure 7:**
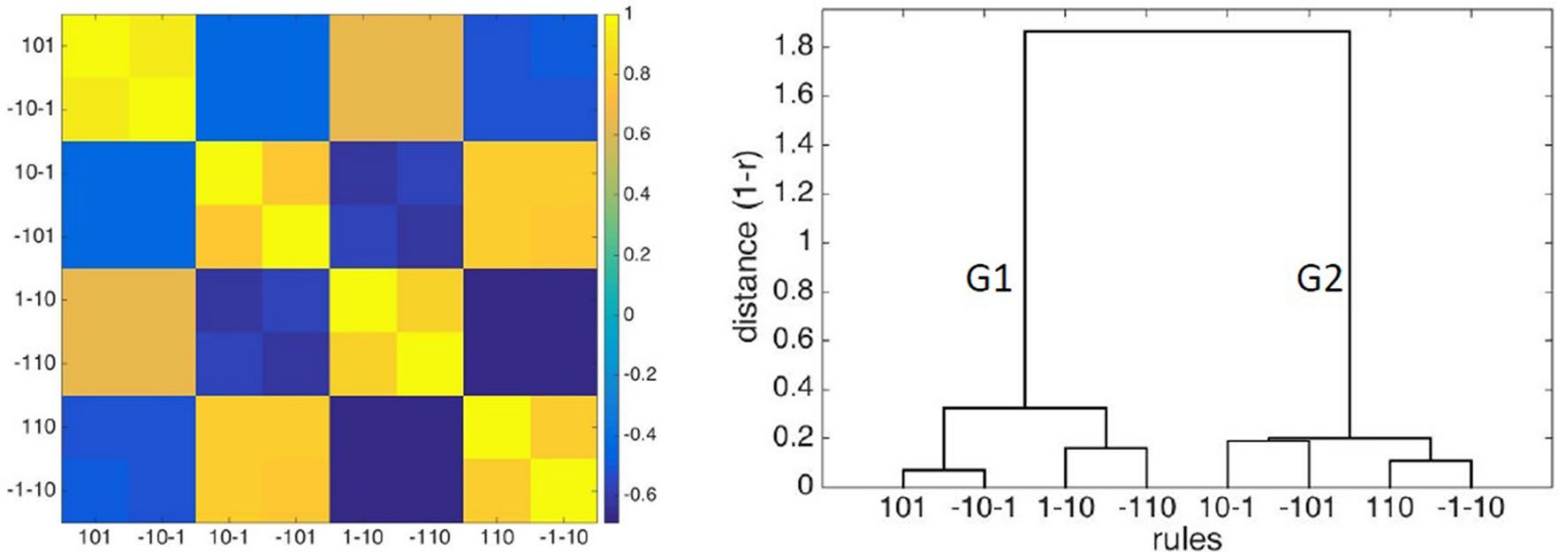
Classification of functional rules. Left panel: correlation matrix among rules (Pearson C.I. scale on the right axis). Right panel: dendrogram produced by a hierarchical cluster analysis. In the hierarchical structure symmetrical rules are clustered: (**a**) In the same group in the first level: (101, − 10–1), (1–10, − 110) (10–1, − 101), (110, − 1–10); (**b**) again in two groups in the second level [(101, − 10–1), (1–10, − 110)]; [(10–1, − 101), (110, − 1–10)]. Rules grouped in the second level appear anti-correlated in the correlation analysis of the left panel).

In order to characterize the eight rules selected in Table 1, we explored their interaction pattern shown by the correlation matrix in Fig. 7 (left panel), from which the following considerations emerge: 1) Rules having a similar functional meaning, although with a different node state, appear more related to each other. This points to dynamical processes operating in the frame of different sets of states; 2) positive and negative relations may exist among particular sets of rules. Figure 7 (right panel) shows a hierarchical classification splitting the eight rules into two groups at the first level, which are further split into four pairs of rules, matching the anti-correlated rules of the previous analysis. Taking into account the two related forms of each pair the following points can considered:

- G1) A_n_ (1) + B_n_ (0) → B_n+1_ (1) and A_n_ (1) + B_n_ (− 1) → B_n+1_ (0) have the functional meaning of (de)activation and "turn off", respectively. In the former case A (de-) activates B to its own state, while in the latter A turns off B if they are in the opposite state;
- G2) A_n_ (1) + B_n_ (0) → Bn_+1_ (− 1) and A_n_ (1) + B_n_ (1) → B_n+1_ (0) have, again, the functional meaning of (de-)activation and "turn off", respectively. In the first case A (de)activates B to the opposite than its own state, while in the latter A turns off B if they are in the same state. Notice, the definition of "turn off" is used to distinguish a process pointing to a de-activation (− 1) from the process pointing to a null-activation (0). Concerning the logical relations: one pair of rules (G1) concern a transition of B to the same state of A (0 → state A); another pair (G2) describe a transition of B to the opposite state of A (0 → non-A state).

Table 2 shows the grouping of rules in line with the above sketched functional criteria:

- ActS = (de-)activates B to the same state of A;
- ActO = (de-)activates B to the opposite state of A;
- TfS = turns off B if A and B are in the opposite state;
- TfO = turns off B if A and B are in the same state.

**Table 2:** Proposed functional classification of the rules.

Function	G1	G2
(De-) Activation	A_n_ (1) + B_n_ (0) → B_n+1_ (1) A_n_ (− 1) + B_n_ (0) → B_n+1_ (− 1) *ActS*	A_n_ (1) + B_n_ (0) → B_n+1_ (− 1) A_n_ (− 1) + B_n_ (0) → B_n+1_ (1) *ActO*
Turn off	A_n_ (1) + B_n_ (− 1) → B_n+1_ (0) A_n_ (− 1) + B_n_ (1) → B_n+1_ (0) *TfS*	A_n_ (1) + B_n_ (1) → B_n+1_ (0) A_n_ (− 1) + B_n_ (− 1) → B_n+1_ (0) *TfO*

The significant rules show a specific nested structure and possible functional role as defined by the four relations: (de-)activation, turn off, same state and opposite state. The two-by-two combination of these features (see the text) point to a functional process described by an appropriate interaction matrix.

In other words, we have a double classification for such rules: a) a simpler process of changing the target node (activate or turn off), and b) a more comprehensive process pointing to uniformity or differentiation within the system, namely to a converging (ActS and TfS) or diverging (ActO and TfO) state among nodes. It is noticeable that the G1 and G2 rules refer, respectively, to the positive and negative functional connectivity and tend to be expressed in different and non-overlapping set of links.

### The multiplex network

**Figure 8:**
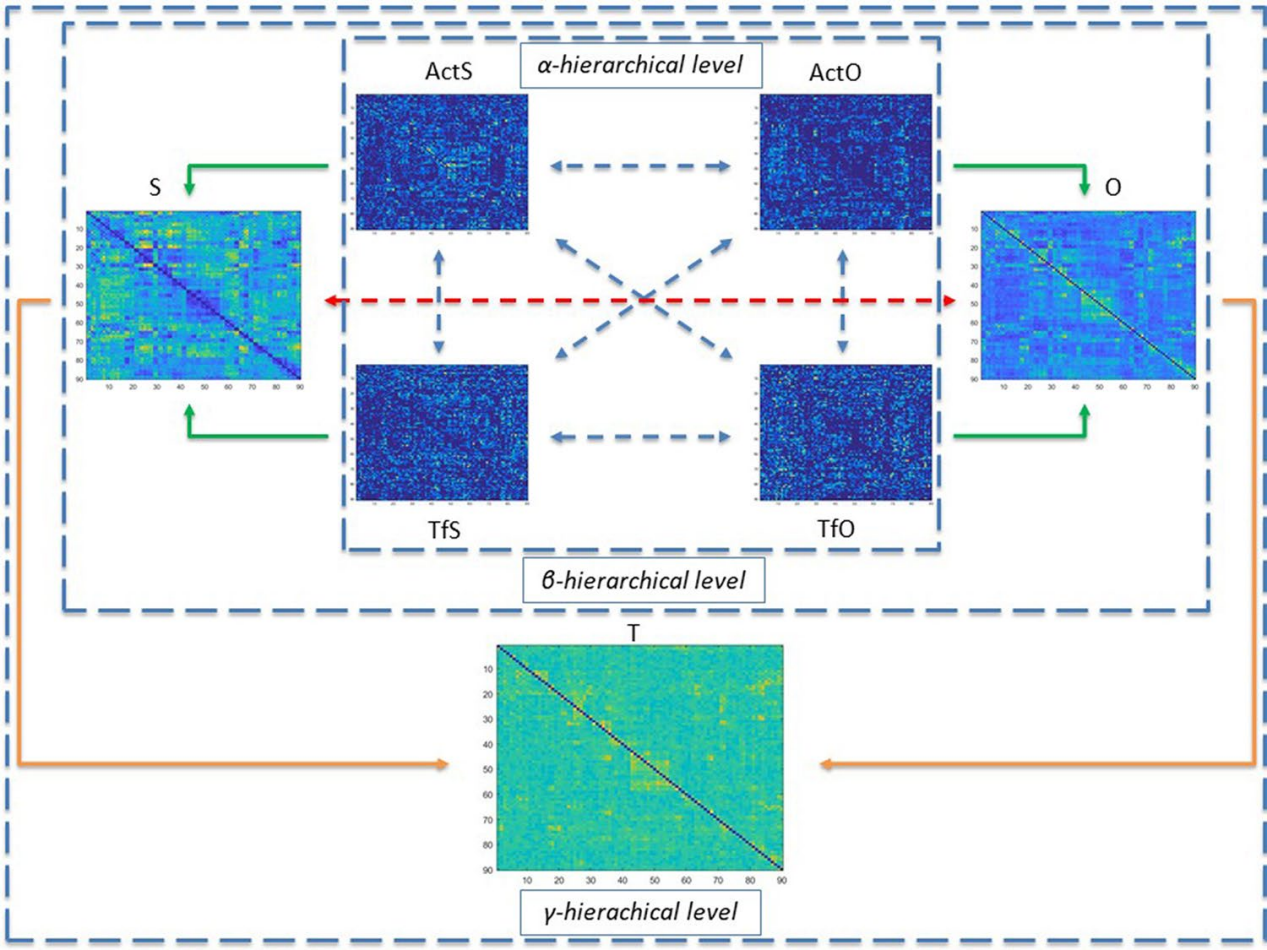
Schematic overview of the multiplex network defined in this paper. The four α-hierarchical level matrices (ActS, TfS, ActO and TfO) are in the central core; the β-hierarchical level matrices (S and O) on the left and the right side of the core, respectively, and the γ-hierarchical level matrix (T) on the bottom side. The interactions among single-layers of the vector of adjacency matrices at the α- and β-hierachical level, are indicated by blue and red dashed double arrow, respectively. The weighted aggregated overlapping adjacency matrices to the higher hierarchical level are indicated by green (from the α- to β-hierachical level) and orange (from the β- to γ-hierachical level) arrows.

The four matrices ActS, ActO, TfS and TfO correspond to the main four layers in the vector of adjacency matrices of a multiplex network, hereafter defined as α-hierarchical level. We extended the analysis to two matrices calculated as weighted, aggregated, overlapping adjacency matrices from those in the α-hierarchical level, namely S matrix (Same state, combination of ActS and TfS), and O (Opposite state, combination of ActO and TfO). The new vector of adjacency matrices was defined in a β-hierarchical level. Finally, a T matrix was calculated as the weighted aggregated overlapping adjacency matrix from S and O matrices, corresponding to a linear combination of the first four layers and which defines the γ- hierarchical level. Figure 8 contains a global representation of the multiplex network including the above-mentioned hierarchical levels.

In Table 3 the characteristic indexes of each layer in the considered hierarchical levels are reported. The T matrix, containing the whole set of weights, appears almost completely connected. In the α-hierarchical level, ActS contains the larger amount of TE values compared to ActO matrices, while the TfS and TfO show the lowest (and almost the same) values. Similar results are found for the connection density. Consequently, the S rule has higher values in both weights and connection density as compared the O rule.

**Table 3:** Descriptive statistics of layers in a multiplex network.

Hierarchical level	Layer	Weight	Connection density
α	Acts	56.1 ± 3.5	0.64 ± 0.02
α	TfS	26 ± 1.1	0.57 ± 0.02
α	ActO	40.5 ± 2.8	0.56 ± 0.02
α	TfO	25.9 ± 1.2	0.55 ± 0.01
β	S	82.2 ± 3.6	0.83 ± 0.01
β	O	66.4 ± 3.2	0.76 ± 0.01
γ	T	148.6 ± 4.4	0.99 ± 0.002

The Interaction Rules refer to the layers in the multiplex network represented in Fig. 8. Weights and Connection density values are given as averages ± S.D.

#### Reciprocity and multireciprocity

As a first global feature of the multiplex network we looked at the Reciprocity Index (RI). Table 4 shows the clear difference with the random model (*p* < 0.001) reached for all matrices but for ActS and ActO, as well as for their interactions in the vector of adjacency matrices. In detail, for the α-hierarchical level both TfS and TfO show negative values, indicating an asymmetric behaviour. Similar results are detected for the multireciprocity in the interacting matrices ActS/TfS and ActO/TfO, while opposite results hold for the interacting TfS/TfO, as well as for ActS/TfO and ActO/TfS. An asymmetric behaviour is also found in the β-hierarchical level matrices S and O, but the opposite behaviour for their interaction S/O, as well as for their sum in the γ-hierarchical level matrix T.

**Table 4:** Reciprocity index (RI) in the multiplex network.

Hierarchical level	Layer	RI (mean ± S.D.)
α	ActS	0.01 ± 0.03
α	TfS	− 0.08 ± 0.02*
α	ActO	− 0.01 ± 0.03
α	TfO	− 0.08 ± 0.02*
α	ActS/TfS	− 0.05 ± 0.02*
α	ActO/TfO	− 0.03 ± 0.02*
α	ActS/TfO	0.08 ± 0.02*
α	ActO/TfS	0.13 ± 0.02*
α	ActS/ActO	− 0.01 ± 0.03
α	TfS/TfO	0.05 ± 0.02*
β	S	− 0.06 ± 0.04*
β	O	− 0.05 ± 0.04*
β	S/O	0.09 ± 0.03*
γ	T	0.03 ± 0.02*

Asterisks indicate significant values (*p* value < 0.05, Bonferroni corrected).

#### Subgraph analysis

Looking at the combination of different patterns of links in the Cycle and Flux triads, the following number of subgraphs in the three hierarchical levels were observed:

- α-hierarchical level: 4 Cycle and 4 Flux (0^th^ order), 12 Cycle and 36 Flux (1^st^ order), 8 Cycle and 24 Flux (2^nd^ order);
- β-hierarchical level: 2 Cycle and 2 Flux (0^th^ order), 2 Cycle and 6 Flux (1^st^ order);
- γ-hierarchical level: 1 Cycle and 1 Flux (0^th^ order).

**Figure 9:**
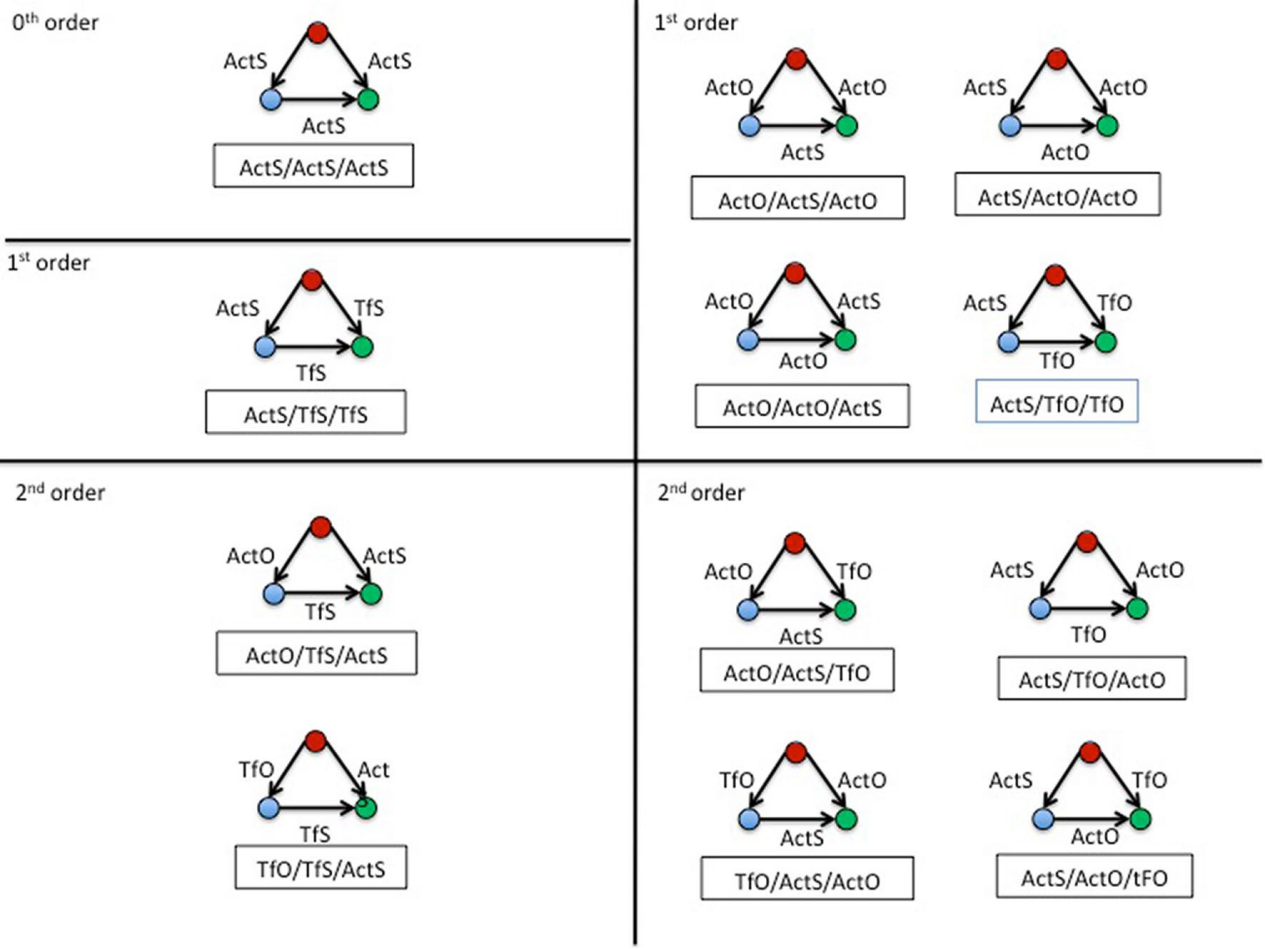
Significant Flux subgraphs of different order. Top left: The Flux subgraph in the ActS layer appear different from random (Flux ActS = I: 1654.2 ± 190.6; *p* value: 6 × 10^−4^ but the coherence does not reach a clear significance (0.03), indicating a low stability; Top right, middle left: Flux subgraphs within ActS and ActO interacting layers appear quite robust, as well as ActS and TfO. ActS and TfS Flux shows a significant I but not C. Bottom left and right: As for the 2^th^ order subgraph in the α-hierarchical level, most of the flux combinations show a significant intensity (I). However, only two reach significance for Coherence (C): Flux ActO/TfS/ActS and Flux TfO/TfS/ActS. See the text for further comments.

A summary of the whole set of possible subgraphs at various hierarchical levels are provided in Suppl. Mat. 4. Figure 9 shows that the whole set of significant subgraphs for both I and C indexes are of the Flux type belonging to the α-hierarchical level, and most of them fall in the 1^st^ and 2^nd^ order. These subgraphs share the same interaction rule acting on the sink (source to sink: ActS; middle to sink: TfS), but differ in the middle way, alternating the two opposite rules: ActO and TfO. Other less stable (not significant C) Flux triads act with the same rules:ActS, ActO, TfO. In the case of the β-hierarchical level, both S and O make a Flux subgraph, while their interaction produces a Cycle subgraph. However, no subgraphs reach a clear significance in both I and C, in this hierarchical level. In Table 5 a complete summary of the above results is reported.

**Table 5:** Intensity and Coherence values of Subgraphs at different hierarchical levels.

Hierarchial level	Order	Subgraph	Intensity (I) mean ± S.D. *p* value		Coherence (C)* P* value
α	1^st^	Cycle ActS/TfO/ActS	1094.7 ± 80.0	0.030	n.s
α	1^st^	Flux ActO/ActO/ActS	1012.0 ± 83.6	4 × 10^−6^*	0.001*
α	1^st^	Flux ActO/ActO/TfS	726.4 ± 64.2	0.01	0.05
α	1^st^	Flux ActO/ActS/ActO	1077.4 ± 89.7	3 × 10^−7^*	5 × 10^−4^*
α	1^st^	Flux ActS/ActO/ActO	1082.6 ± 89.5	3 × 10^−13^*	5 × 10^−5^*
α	1^st^	Flux ActS/ActS/TfS	1136.6 ± 90.6	0.03	n.s
α	1^st^	Flux ActS/TfO/TfO	806.4 ± 50.4	8 × 10^−11^*	2 × 10^−4^*
α	1^st^	Flux ActS/TfS/TfS	855.3 ± 58.0	1 × 10^−4^*	0.04
α	1^st^	Flux TfO/ActS/TfO	795.0 ± 50.3	0.02	0.02
α	1^st^	Flux TfS/ActS/TfS	838.3 ± 57.3	0.03	n.s
α	1^st^	Flux TfS/TfS/ActO	754.0 ± 60.2	0.01	n.s
α	2^nd^	Cycle ActO/TfS/ActS	918.3 ± 44.0	0.0006*	n.s
α	2^nd^	Cycle ActS/TfO/TfS	797.5 ± 44.3	0.02*	n.s
α	2^nd^	Cycle ActS/TfS/ActO	918.3 ± 41.8	0.0003*	n.s
α	2^nd^	Cycle TfS/TfO/ActS	798.0 ± 43.2	0.02	n.s
α	2^nd^	Flux ActO/ActS/TfO	902.7 ± 46.2	1 × 10^−5^*	0.005
α	2^nd^	Flux ActO/TfS/ActS	889.6 ± 43.4	4 × 10^−6^*	0.002*
α	2^nd^	Flux ActS/ActO/TfO	893.7 ± 47.2	2 × 10^−8^*	0.003
α	2^nd^	Flux ActS/TfO/ActO	886.6 ± 46.7	1 × 10^−5^*	0.02
α	2^nd^	Flux TfO/ActS/ActO	896.2 ± 44.8	0.0006*	0.01
α	2^nd^	Flux TfO/TfS/ActS	779 ± 43.5	2 × 10^−5^*	0.002*
β	0^th^	Flux S	3870.5 ± 252.9	0.03	n.s
β	0^th^	Flux O	2877 ± 129.5	0.01	n.s
β	1^st^	Cycle S/O/S	3330.2 ± 117.4	0.02	n.s

Asterisks on the *p* values indicate significance after Bonferroni correction—see the text for details.

#### Centrality features

**Figure 10:**
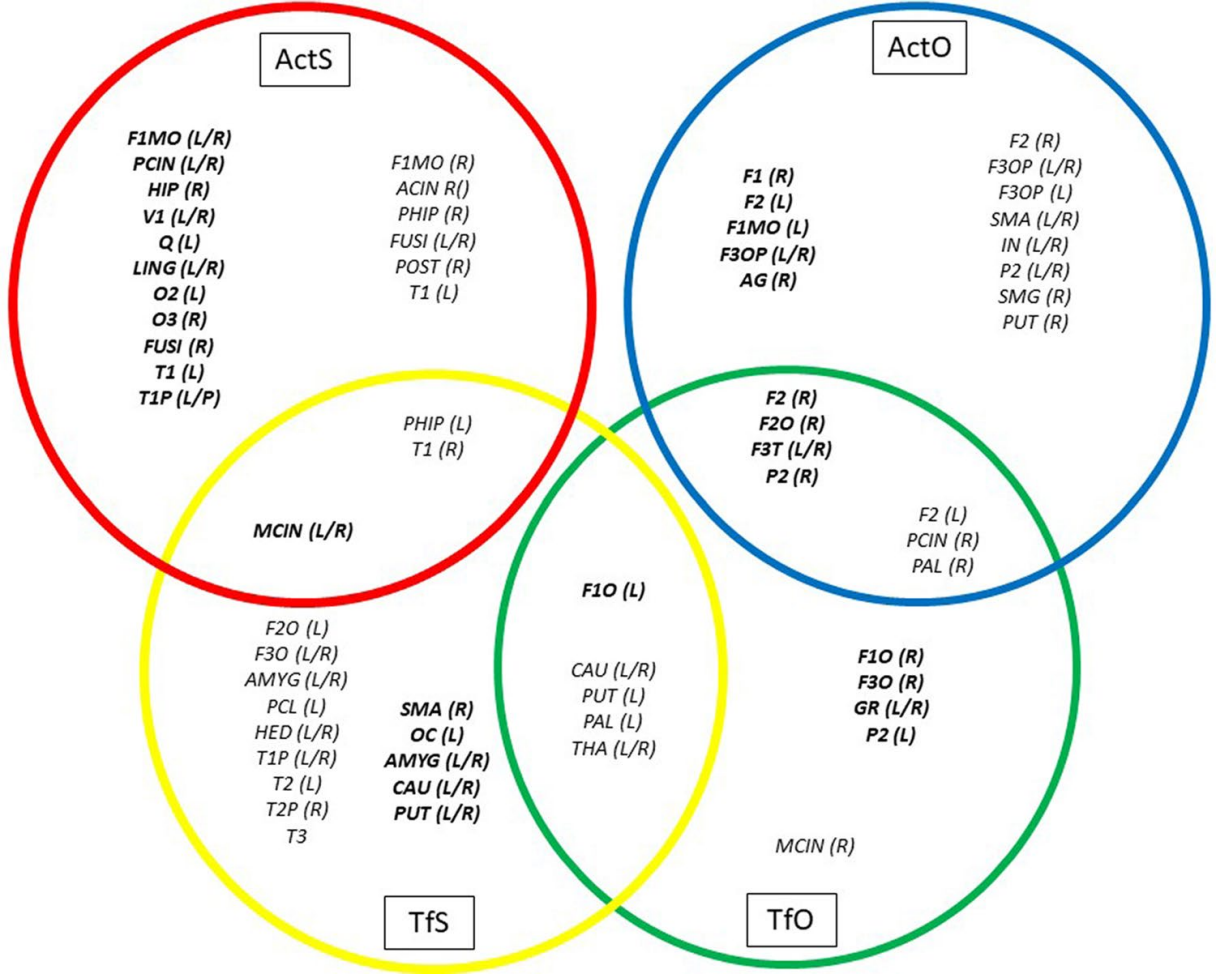
Central nodes in the α-hierarchical level of the multiplex network. The coloured circles are associated to the rules at the α-hierarchical level: red = ActS; orange = Tfs; blue = ActO; green = TfO, and in each circle the brain regions acting as local central nodes are reported. The intermediate multiplex central nodes are in the intersections. In bold are the in-strength central nodes. Notice that: (**a**) the basal ganglia regions are shared by the TfS and TfO rules; (**b**) the fronto-parietal cortex falls within the AcsO and TfO rules; (**c**) a sparse number of brain regions covering the middle cingulum, the parahippocampus and the superior temporal gyrus, are found in the ActS and TfS rules.

The Centrality Index of each layer shows different patterns of different brain areas (nodes) as a function of their in- out-connection weights. In what follows each layer will be separately considered in each hierarchical level. Central nodes of ActS and TfS rules are inserted in Fig. 10, in red (ActS) and yellow circle (TfS), respectively. Regarding the first one, most of the out-strength and in-strength central nodes fall in the vision related brain regions (calcarine, lingual and fusiform) and limbic/DMN areas; the total strength confirms the previous brain areas as the main nodes of this rule. Conversely, central nodes of the TfS rule have a well distinct pattern of in- and out-strength central nodes, mainly centred in the sub-cortical regions of basal ganglia (caudate, putamen, pallidum) and limbic (amygdala). Other hubs of out-strength are found in motor related areas (SMA and middle cingulum) and in the frontal superior gyrus, while in-strength hubs cover a sparse cortical area including temporal, frontal and limbic brain regions.

The nodes of ActO and TfO rules are inserted in Fig. 10 in blue circle and green circle, respectively. Central nodes of ActO cover almost the whole area of the dorso-lateral pre-frontal cortex and the inferior parietal gyrus (parietal inferior, angular, supramarginal). However, in the out-strength also sensory-motor (SMA, insula), sub-cortical (pallidum and putamen) and DMN (cingulum posterior) regions appear as central nodes. The TfO rule has an intermediate pattern between the TfS and the ActO, having the cingulate cortex (anterior and posterior) and sub-cortical regions (thalamus, pallidum, caudate, putamen) as the main out-strength central nodes, and the fronto-parietal cortex as in-strength central nodes.

**Figure 11:**
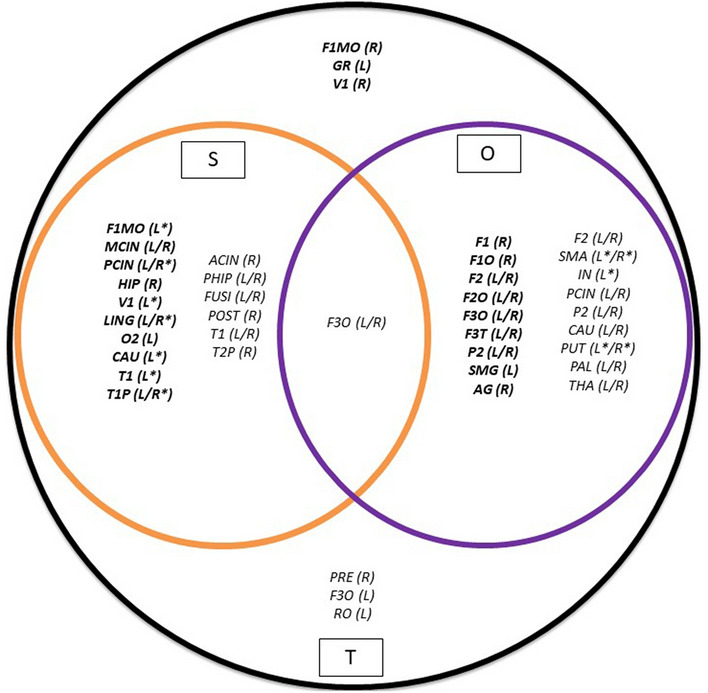
Central nodes in the $\mathrm{\beta }$- and γ-hierarchical level of the multiplex network. The coloured circles are associated to the rules as follows: orange = S ($\beta )$; violet = O ($\beta )$; black = T (*γ)*, and in each circle the brain regions acting as local central nodes are reported. In the intersections are the brain regions acting as intermediate multiplex central nodes. The asterisks indicate overlapping brain regions between the intermediate (β) and the highest (γ) hierarchical level. In bold are the in-strength central nodes. Notice that: (**a**) only in the black circle are localized brain regions that do not appear in the intermediate hierarchical level; (**b**) overlapping brain regions of the α—level disappear in the β—level.

In the β-hierarchical level, Fig. 11 (orange circle: S; violet circle: O) confirms as central regions the sensory-limbic related areas and the fronto-parietal network for S and O rules, respectively. However, less evident appears the sub-cortical action of the TfS and TfO components since the weights of these last rules are covered by those of the ActS and ActO rules. More intriguing is the result at the higher hierarchical level of the T matrix (Fig. 11, black circle), where the main out-strength central nodes are detected in a sparse brain region (sub-cortical, motor and attention networks), and the same holds for the in-strength ones (sensory-related areas, limbic and sub-cortical regions).

In conclusion, looking at the results of the α-hierarchical level (corresponding to the vector of adjacency matrices), several nodes appear in more than one layer: intermediate multiplex nodes (two layers). In particular, the basal ganglia regions (caudate, putamen, pallidum and thalamus) appear to be shared among the TfS and TfO rules as out-strength central nodes, as well as in the total strength. Thus, these sub-cortical regions appear as a possible intermediate station for the modulation of the Turn Off rules. Moreover, the ActO and TfO rules taken together cover most of the prefrontal and parietal cortex for outward connections. As a matter of fact, the O rule seems having a major role in these brain areas. Finally, the ActS and TfS rules share the cingulum middle, as an out-strength central node, and the parahippocampus and the temporal superior gyrus as in-strength central nodes, respectively. Again, these results are confirmed by the central nodes found in S rules of the β-hierarchical level. No central nodes are found between the ActS and ActO rules. In Figs.10 and 11 a summary of local and intermediate central nodes is reported within the corresponding layer. A more detailed account of single connections is included in Suppl. Mat. 6.

#### Modularity

When compared to the random model, only ActS and S rules show a significant increment of Q values (Table 6). In both rules the same three main modules were found (Table 7): the first one referring to the visual cortex and its process-related areas; the second one including the Default Mode Network (DMN) and limbic regions; the last one covering most of the Task Positive Network (TPN) and including dorsolateral prefrontal cortex, parietal cortex, basal-ganglia, auditory cortex and sensory-motor cortex.

**Table 6:** Q index comparison between static and dynamic modularity. Only ActS and S rules show a significant increment of Q values (*p* < 0.001) when compared to the random model.

Hierarchical level	Rule	Q
α	ActS	0.11*
α	TfS	0.004
α	ActO	0.007
α	TfO	1*10^−16^
β	S	0.07*
β	O	0.003
γ	T	0.004

**Table 7:** Modules of ActS rule.

Module 1—visual	Module 2—DMN/limbic	Module 3—TPN & other
V1	F1	PRE
Q	F1O	F2O
LING	OC	F3OP
O1	F1M	F3T
O2	F1MO	F3O
O3	GR	RO
FUSI	ACIN	SMA
	PCIN	IN
	HIP	MCIN
	PHIP	AMYG
	AG	POST
	PQ	P1
	T2	P2
	T2P	SMG
	T3	PCL
	CAU (L)	PUT
		PAL
		THA
		HES
		T1
		T1P
		CAU (R)

The network includes three main clusters: the visual cortex; the limbic/DMN; the sparse brain regions belonging to the TPN. Unless explicitly indicated, all modules refer to both (L/R) hemispheres.

A specific left/right asymmetry is detected in the caudate: left and right split in DMN/limbic and in the TPN module, respectively. We hypothesize that the caudate tend to get connected with both modules, as a sort of bridging node. Note that since this analysis concerns averaged matrices, the networks obtained is fully connected, and the combinatorial randomization was used.

## Discussion

In order to explore causal influences between brain areas a data-driven approach is proposed, using fMRI data under resting state and taking advantage of the Transfer Entropy (TE) concepts. In the Suppl. Mat. 1 and 2 a flow chart of the adopted analytical strategy as well as the brain areas (ROIs) used in this paper, are included. As compared to previous works in the field^1,7,8^, we describe a new way to estimate the functional brain causal interactions by TE parameters somehow similar to the Granger Causality^7,47^. At difference with the latter method, however, we use a non-parametric approach and avoid a whole set of a priori statistical assumptions. Another and most popular method is the Dynamic Causal Modelling^1^ (DCM), based on a series of differential equation arranged in a hemodynamic model^46^, a recent updating of which has been proposed for the resting state condition^4^. The above-mentioned models, however, do not consider the different kinds of brain interactions although a recent work of Zhou et al*.*^17^ focused on negative interactions looking at feed-back (negative) and feed-forward (positive) connections.

### Transfer entropy (TE) parameters

The TE estimate was initially proposed by Vicente et al*.*^48^ to characterize brain causal interactions. In our case we use a discrete time-series analysis where continuous values in a time-series are converted into a finite number of discrete events by threshold filtering the amplitude of BOLD signals. This allows the detection of cerebral states closer to the temporal resolution of the data acquisition with no (or few) assumptions and no substantial waste of information. In this respect Tagliazucchi et al*.*^31^ showed that only considering spontaneous events the resting-state network can be recovered by a continuous signal fluctuations method. In addition, Petridou et al*.*^49^ deconvolving a hemodynamic response function in the resting state, clarified how spontaneous events may contribute to the correlation strength and the power spectra of slow spontaneous fluctuations. We found four different kinds of interactions among brain areas, which by hierarchical clustering can be reduced to two sets sharing similarities with positive and negative functional connectivity and in which two alternative mechanisms are active: (de)activation and turn-off. A particular modulation of brain areas emerges including not only activations and de-activations but also a subtle mechanism of turning off. The individual variability of the observed events was checked by a statistical analysis on each subject. All positive interactions (ActS and TfS) were validated on the whole data set, while the negative ones (ActO and TfO) seem less accurate. This agrees with the higher variability of anti-correlations in 0-lag correlations estimates of brain connectivity^13^. A possible explanation is the different synchronization timing for the negative connectivity^16,50^. A more speculative proposal invokes a faster transfer mechanism associated to negative interactions. Exploring the variability changes as a function of different levels of time-lag connectivity would provide additional evidence to that. In our approach the distinction between positive and negative functional connectivity is a leading idea and our results, although preliminary, could open the door to a reliable detection of clinical differences among individuals in different cognitive states. In this regard, to compare functional and causal brain connectivity, as for the Dynamic Causal Modelling (DCM) estimation, the bivariate correlation appears the most natural method to study the hypothetical relation between the brain states transition and the causal brain interaction^5^, the CAP approach^3^, using a discrete method to analyse the BOLD signal, would be our favoured one.

### Functional interpretation of the rules

Assuming that brain states can be estimated by the fluctuating combination of node connections^51^ defining a functional connectivity state or meta-state, a particular cognitive condition could be characterized by a finite set of meta-states^52^ where different connectivity patterns alternate^53,54^. As a matter of fact, several evidence showed how these dynamics draw a phase transition between more or less integrated/segregated network structure^55^. Brain dynamics are usually defined in terms of meta-stability^56^ or multi-stability^57^ in the dynamical system frame. Both terms point to a fluctuating dynamic between different sets of synchronized states as a possible explanation of functional flexible (in)stability, (see Breakspear 2017^57^ for a general review). In such a frame, the four rules we found provide an exhaustive description of the network dynamic: activation rules change the node states from 0 to 1 (or − 1); the turn off rules gets back to 0 the node state. Both the ActS and TfS rules promote some uniform node states: the former one changes the target node to the same state of the source, the latter turns off the target node if in the opposite state of the source. Conversely, the ActO and TfO rules promote a process inducing the activation to an opposite state than the source node and turning off nodes having the same state than the source node, respectively. In brief, these rules can describe a self-sustained dynamic, following an initial condition and perturbed by external or internal stimuli.

Assuming that the state of co-activated brain regions can be driven by causal interactions (ActS) while the mutual de-activation can be maintained by the ActO rule, both ActS and ActO may share a similar physiological interpretation^10,11,58^.

Conversely, the turn off rules do not seem to overlap any of the functional measurements in use.

From a neurophysiological point of view, such interactions could be associated to two functions: 1) turning off a co-activated set of brain regions; 2) enhancing the excitability of brain regions. The former function arises when a particular action has to be suppressed, the latter function puts the node in the condition to be changed to an alternate state (notice that 1 or − 1 cannot be changed without passing from 0). Thus, the rule has both abilities: turning off a functional state of a brain region and enhancing the excitability of the region towards interactions. Such a mechanism could be related to the inhibition of a tonic depolarization, for example the action of a GABAergic interneuron. However, these considerations remain speculative at this stage and to check the above sketched dynamical features appropriate simulations should be performed, e.g., in some agent-based simulation environment^59^.

In summary, a specific synchronization of action rules within a given geometrical network could generate different dynamical patterns, and each change of nodes' state can be driven by the network connection itself, without necessarily needing an external or internal disturbance.

### Network analysis

A multilayer network was analysed on the asymmetric adjacency matrix of each subject. This matrix describes the network of the corresponding interaction rule and accounts for the 8100 possible interactions between the 90 brain regions. Three hierarchical levels have been considered as indicated by the hierarchical clustering analysis (Fig. 7), as well as all the possible inter-layer relations. It appears that the most significant features characterize the lower hierarchical level only, while merging the information in a higher hierarchical level seems to mitigate (or dilute) the specific trait of the single rule network in the lower hierarchical level. The four basics rules show peculiar features characterizing each of them in a specific manner.

In dealing with the dynamic exactly occurring between brain regions a main problem for us was the lack of knowledge concerning the real timing of events in the reciprocal connections as well as triads. Although we know that rules tend to be arranged in a particular structure, we ignore whether the links work together (are synchronous), or in sequence. However, we can infer the possible temporal sequence by studying the logical relations among rules. Thus, a particular combination of links can be inferred, and the relative dynamic predicted. For example, in the case of triadic subgraphs the links from the source node could be synchronous, while the connection from the middle to the sink node could work afterwards: on the sink node cannot insist at the same time two links endowed with the Turn Off (TfS or TfO) and the (de-)activation (ActS or ActO) rules, because they need a different state on the node (1 or − 1 state for the Turn Off rules and 0 for the De-/Activation rules).

#### Reciprocity and multireciprocity

As a former comment it can be noted that the brain networks show a not reciprocal nature, most marked in the turn off rules. The same is obtained for interacting matrices in the same kind of process (ActS/TfS and ActO/TfO), indicating a preferential unidirectional flow of interactions within the same functional rule. However, reciprocal connections arise between opposite interaction processes, pointing to a possible feedback mechanism. Similar relations are found for the higher hierarchical level S and O, while in the T matrix the network lost the single layers detail becoming almost reciprocal.

Concerning the dynamic, the links from two biunivocal nodes can be synchronous or in sequence as a function of the state availability of the node B_n_ towards the state of the node A_n_ and vice versa. The interaction rules with a significant reciprocal connectivity are: a) TfS/TfO, b) ActS/ActO and c) ActO/TfS. In the first case TfS and TfO rules cannot be expressed at the same time since the combination of state (same and opposite state, respectively) can work in one direction only, same consideration concerns the sequence hypothesis (the B_n+1_ state, necessarily 0, is not available for the turn off rules). Thus, a timing dependence action cannot be defined between these reciprocal connections. In the second and third cases a synchronous action is not possible since 0 state, required for the node B_n_ in the (de-)activation rules, does not correspond to a possible state of the node A_n_ for any kind of rule. Conversely, a particular sequence interaction is possible, and a hypothetical feed-back mechanism can be defined (see Suppl. Mat. 5).

By our results, the ActO rule is not characterized by a reciprocal connection (n.s.), indicating a low probability of bi-directional connections. In this regard, we hypothesize that the mutual deactivations are expressed by different hubs of TPN and DMN connecting different non-hub regions, in terms of crossing but not overlapping structures. This architecture would be in line with the model proposed by the network analysis of the (static) negative brain functional connectivity^13^, and possibly confirmed by the single links analysis in Suppl. Mat. 7 and commented in the following Sect. (4.3.3 Cerebral circuits).

#### Subgraphs

**Figure 12:**
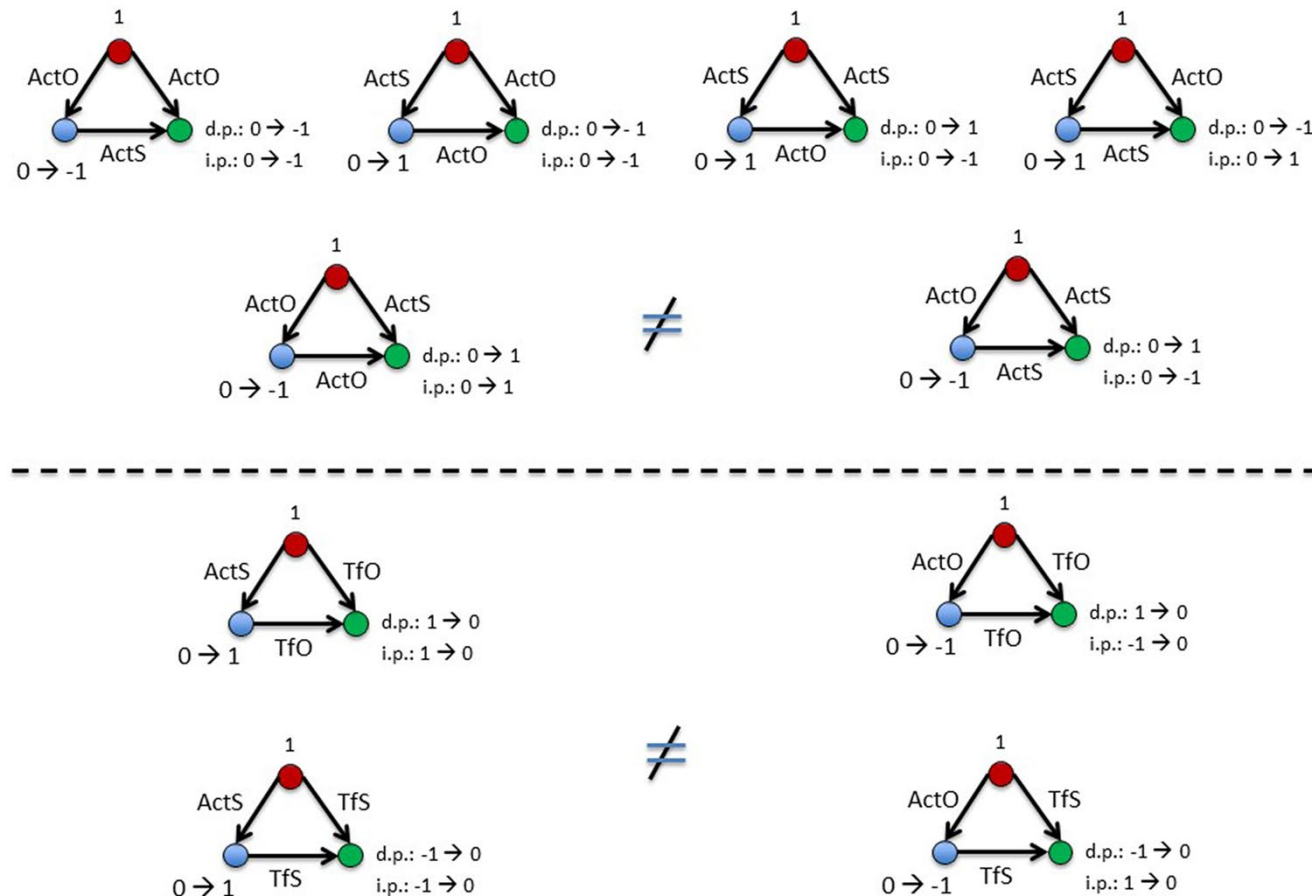
Dynamic of 1^st^ order subgraphs. Source, middle and sink nodes are in red, blue and green colour, respectively. From the source node two pathways can be characterized: an indirect pathway (i.p.) passing through the middle node, and a direct pathway (d.p.) going directly to the sink node. The time-dependent changes of the nodes state are reported for both indirect (i.p.) and direct pathways (d.p.). The triads in the left and right sections are not isophorms. For the difference between top and bottom panels see the text. Links can work in a synchronous or sequential way depending upon the relative state of the interacting nodes.

The proposed dynamic of the 1^st^ order subgraphs are reported in Fig. 12. The three cases in the top right panel of the figure point to the same (de-)activated state between the source and the sink node. At the same time, the last two (bottom right panel) result in the same null (0) state. We asked whether these properties are shared by other combinations of subgraphs and, by the same procedure on the other 1^st^ order subgraphs (left panel), we checked that these triads are unique. Thus, taking into consideration the possible logical temporization, we hypothesize two general dynamic: 1) a switch two pathway for the same target with different temporization (direct and indirect pathway); 2) a reinforce pathway to have a synergic action on the sink node.

**Figure 13:**
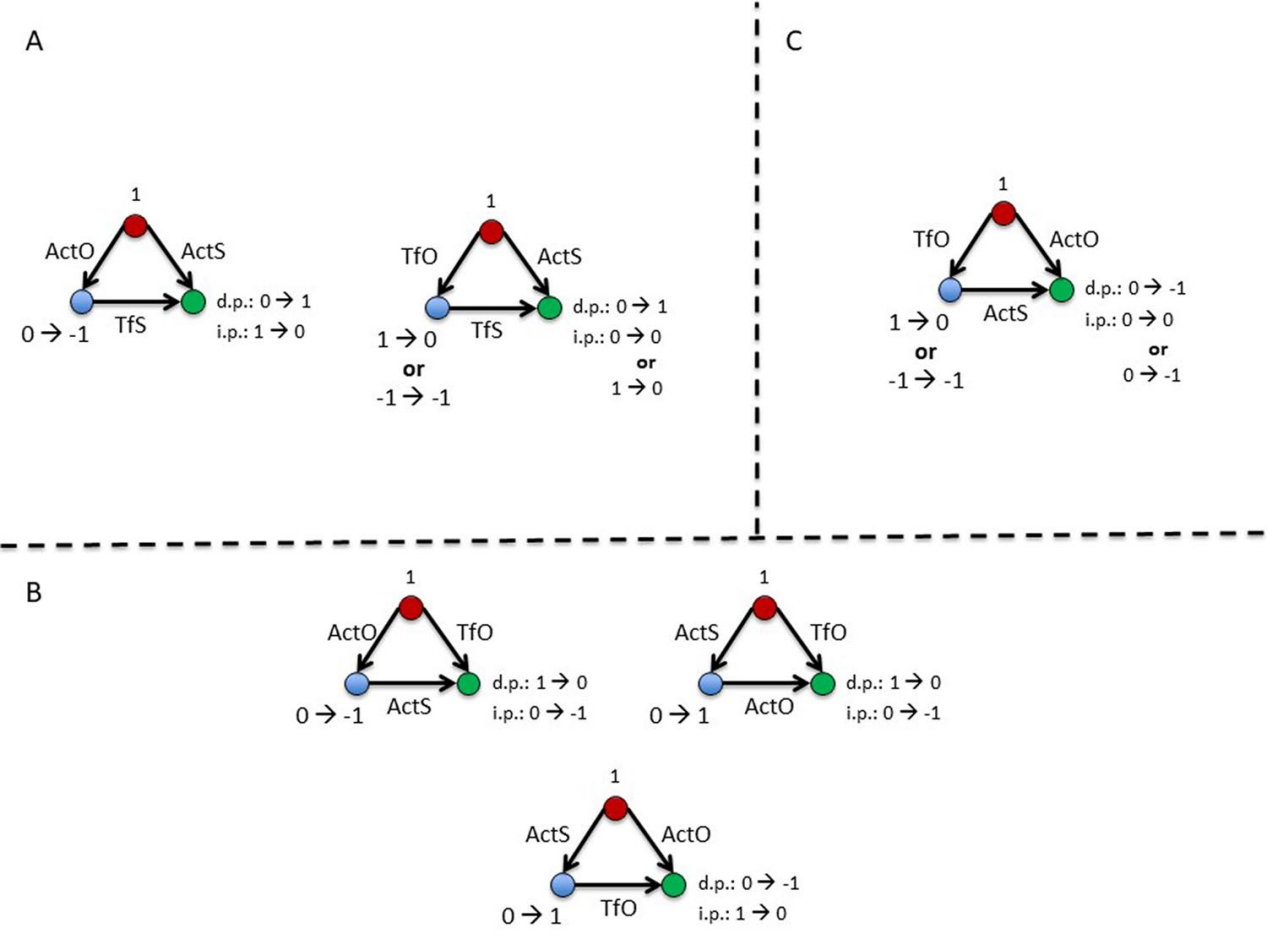
Dynamic of 2^nd^ order subgraphs. Source, middle and sink nodes are in red, blue and green colour, respectively. (A) On the sink node the ActS rule applies on the direct pathway, and the TfS on the indirect pathway. The middle node is modulated by an opposite rule: ActO and TfO for the ActO/TfS/ActS and the TfO/TfS/ActS triads, respectively. (B) the ActS/TfO/ActS and Acts/ActO/TfO, are symmetrical Flux triads in which the same mechanism operates: indirect pathway (de-)activates the sink node in the opposite state than the source, while the direct pathway turn off the sink node if in the same state of the source. The same occurs, although with inverted pathways, for the Flux ActS/TfO/ActO, in which the indirect way turns off the sink node if in the same state of the source, while the direct way changes its state to the opposite than the source. (C) The Flux TfO/ActS/ActO operates as a competition solving mechanism: since the sink node is subjected to the competing rules ActS and ActO, the source to middle pathway solves the impasse turning off the ActS rule and promoting the opposite state. It is worth to note that also in this case the symmetrical form promoting the ActS rules is not significant.

In the case of the 2^nd^ order subgraphs, a more complex situation emerges including the remarkable conditions depicted in Fig. 13, in which: the source node seems to operate by two alternative ways on the sink node (activated or turned off) modulating an intermediate reaction in the indirect pathway (top left panel). Moreover, a whole series of fine modulations seems to occur along the direct and indirect pathways characterizing most of the information transfer mechanisms of the considered phenomena (top right and bottom panels). As a summary conclusion, it appears that the brain dynamic promotes a non-competitive behaviour in both first and second order subgraphs. This is quite reasonable to overcome possible conflicting situations. Moreover, the mechanisms are arranged in a hierarchical way: in the first order (two layers) only the congruent triads, for the direct and indirect pathways, are promoted; in the second order a possible compensatory mechanism (feedforward) appears. This suggests that the complexity of the brain dynamic emerges when more than one connection type is considered. However, merging the rules in the higher hierarchical levels may lose the specific information on the interaction of single layers and hide significant results.

At our knowledge, we report for the first time a subgraph analysis in a multiplex network derived from an fMRI dataset. Other papers studied a multiplex brain network using MagnetoEncephaloGraphy (MEG)^60,61^, but due to the quite different approaches we did not try a critical comparison with those results. In a previous works carried out using fMRI datasets, not in a multiplex perspective, the close triangle motifs are lacking, and the open motifs are detected as significant subgraphs^62^. The same was found in a classical paper on anatomical connectivity by Sporn and Kotter^63^. In our work no triads are detected in the single layers (but for a trend in the case of ActS). Thus, it is possible to consider that the open motifs could characterize the single layer, while the triad motifs may arise only when more than one layer are taken into consideration. Furthermore, we did not find significant triangle subgraphs at higher hierarchical levels and hypothesize that in a general estimation of only directional connections the role of the possibly different connection pathways is hidden. In the same way, in the anatomical connectivity a functional characterization of connections is lacking, and thus important information about the system is lost. It is worth mentioning that a comparison between anatomical and functional connectivity in a multiplex approach has been only recently proposed^64^.

#### Cerebral circuits

On the basis of our results, the following general considerations concerning a possible cerebral circuit including the four rules can be drawn:

- the ActS rule can play a major role in the intra-module cortico-cortical connections and in carrying perturbing stimuli both external (vision) and internal (limbic / DMN areas);
- the fronto-parietal network and the DMN show a reciprocal, but non-overlapping, deactivation dynamic using the ActO rule;
- sub-cortical regions (mainly basal ganglia) are connected with large cortical networks using the turn off rules (TfS and TFO) and appear as possible intermediate stations of brain cortex modulation.

**Figure 14:**
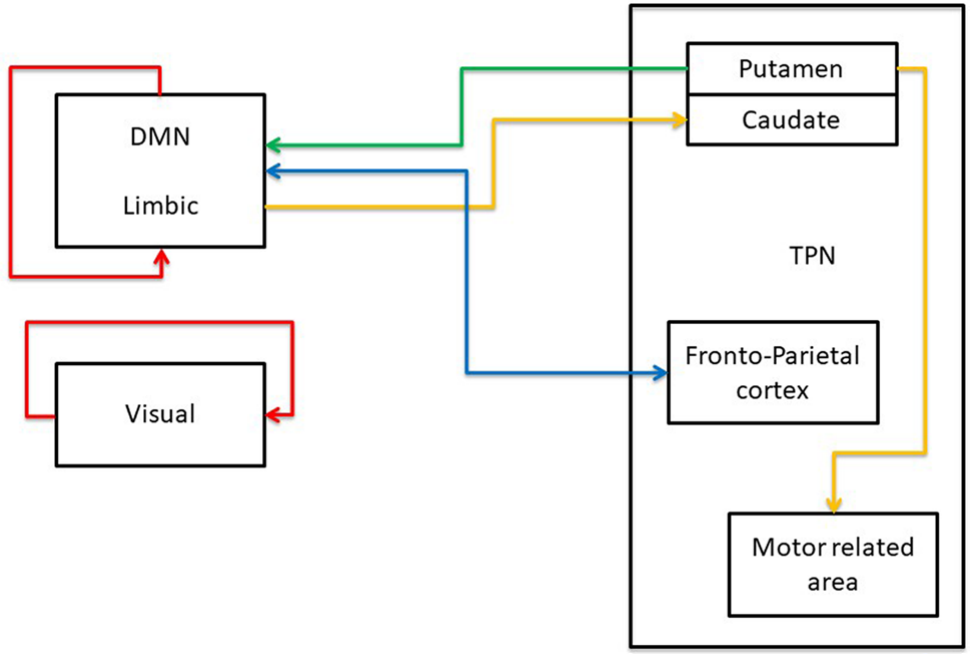
Minimal scheme of brain circuits. Integrating the single nodes link information and the centrality analysis within the network modularity architecture, different patterns of brain areas are associated to a specific rule (for details see ‘Centrality and modularity analyses’ section). Red line = ActS; orange line = Tfs; blue line = ActO; violet line = TfO.

A schematic overview describing this circuit is reported in Fig. 14.

The analysis of the brain functional multiplex network allows a comprehensive comparison with previous works.

As for the ActS rule, the DMN/limbic and vision related areas are the main out-strength and in-strength nodes. At the same time, the modularity analysis shows a significant subdivision of the network into more connected modules: vision, DMN/limbic and sparse regions of TPN. These results point to a possible recursive nature of that rule.

A last consideration concerns the possible role of the ActS rule as source of internal/external perturbation. If the experimental design deals with the resting state, it should not be surprising that the main ActS nodes fall in that state. As a matter of fact, subjects are open-eyes and free to perceive visual stimuli. Moreover, since they are not engaged in any task, other main perturbations may arise from internal stimuli. The main nodes fall, in fact, in the processing visual perception, as well as in the limbic and DMN brain networks, commonly activated in the rest condition^65^.

It is quite interesting that the ActO rule appears associated to the pre-frontal brain regions which, according to previous evidence, are the main hubs of an anticorrelated network^13^. These regions have been recently associated to a causal interaction suppressing the DMN activity^66^ while previous studies identified, by a DCM approach^17^, the dorsal attention network (superior parietal lobe, FEF and inferior frontal gyrus) and the salience network (anterior cingulate, anterior insula and prefrontal cortex) as inhibitory nodes on the DMN, but not vice versa. In such a context, we found that the same prefrontal and parietal regions appear as a main in-/out-strength station, endowed with both active and passive roles.

Studying in deep the single connections, in the TPN/DMN competitive behaviour a large amount of connections involve the DMN hubs (frontal medial orbitalis and cingulate posterior) and a number of frontoparietal brain regions (frontal inferior, SMA, insula, parietal inferior and supramarginal), while the lateral frontal cortex (frontal middle and inferior) is deactivated by the medial part of the same lobe. Similar results are produced by studies of causal connectivity estimated by Granger Causality^7^. Although, in general, different results may be due to different causal estimation methods, the Granger Causality, a data-driven approach based upon time-delay signals, is more in line with our method. As a matter of fact, previous works argued that the TE results coincide with those of Granger for Gaussian variables^67^. In this regard, since the large amount of variable to check (90 ROIs for 180 subjects) and the sample variability, some of those may satisfy the statistical premises of Granger, pointing to similar results. Such a results, however, could be ascribed to some technical details more than physiological issues, not allowing a direct comparison between the two methods.

Finally, taking into consideration both turn off rules (TfS and TfO), the basal ganglia circuit appears as the most involved in the modulatory interactions above. According to structural and functional evidence about basal ganglia in human and non-human primates^68,69^, the main cortico-striatal connections are located in the prefrontal, motor and limbic cortical areas. In our results the putamen modulates the motor related areas (SMA, pre-central, rolandic operculum) using the TfS rules, and the limbic (rectus) and the DMN (cingulum posterior, precuneus, angular gyrus) using the TfO. As for the caudate, a high number of connections come from the medial/orbital frontal regions of the DMN (anterior cingulate, the frontal superior medial and medial orbitalis gyrus) and the limbic network (parahippocampus, the olfactory cortex and the rectus) only with the TfS rule.

It is worth remembering that the basal ganglia are topographically differentiated on the basis of their cortical projections in the following way^70^: the most rostral and ventral part are connected to the medial and orbital frontal regions; the central and dorsolateral parts of putamen take connection with the primary and supplementary motor cortices; the dorsal caudate is connected to the dorsal prefrontal and parietal regions, while the posterior part of the caudate with the posterior lobe (including temporal, parietal and occipital cortex). Recent functional studies in humans confirmed this structural evidence using fMRI data^71^. In our work we could not distinguish the anatomical subdivision of putamen and caudate and check the precise anatomical location of the connections. However, the cortical topographic differences resulting from our data were obtained by measuring different kinds of connections. Thus, it is not possible to exclude that cortico-striatal functional connections could depend upon different kinds of dynamical interactions. The detailed mapping of such interactions, a demanding experimental and modelling endeavour, deserves priority in our future work.

### Limitation and future prospects

The major limitations of our study concern:

- the low time resolution of fMRI data^72^, such data, in fact, have no well-defined time resolution, and spurious results can be easily obtained. Fast fMRI acquisition^73^ would be a possible solution to the problem.
- the resting state paradigm, in such an experimental condition subjects are not checked for a particular request, and less robust results can be obtained. In order to overcome this point, in a further step of our study comparison between different states will be explored.
- the spatial resolution, as a matter of fact, we used a not-so-recent atlas^28^ (AAL) to compare our results with our previous work. Taking into consideration that different brain atlas with different spatial resolution could give different results, an in-depth analysis of the causal connectivity as a function of spatial resolution is also in our research plans.

## Conclusion

Recently developed multivariate statistical methods allow to model brain fMRI signals in a large-scale network where brain regions can be associated based on their functional behaviour. We report on a systematic study of the causal relations in information transfer mechanisms between brain regions under resting condition. Keeping in mind the bulk of problems which affect, in general terms, any causal inference model of fMRI data^5^, we tried to address them by an approach extending the feedforward algorithms and including—among other things—cyclic graphlets in a model-free and data-driven method.

We first introduced a new method to estimate effective connectivity from brain functional data and found four kinds of significant brain interactions (rules). Then, the complex interaction patterns of brain regions were studied in the frame of the multiplex network analysis characterizing the brain network properties at both dynamical and structural levels. In the first case a feedback and feedforward mechanism are inferred characterizing the dyadic (reciprocal) and triadic (subgraphs) network structure. At the structural level, a particular pattern of brain regions can be characterized by a different kind of functional interaction, pointing to a general description of possible cerebral circuits within a brain modular architecture. Thus, we describe for the first time a multilayer (multiplex) network derived from a causal estimated relation on fMRI dataset.

Moreover, by hierarchical clustering methods the four rules can be reduced to two sharing some peculiar (anatomical and functional) properties with positive and negative functional connectivity and suggesting possible common neurophysiological mechanisms.

The research strategy followed in the paper has been compressed in the form of flowchart and reported in Suppl. Mat. 1 while Fig. 14 depicts a possible cerebral circuit including the four rules.

Finally, since our model stems from a dataset of healthy subjects without a specific phenotypic characterization and in a resting state condition, a systematic evaluation of possible different brain connectivity patterns would be precious in different experimental conditions, as well as within different phenotype subjects.

## Supplementary Information

Supplementary Information.
