# Supplementary material for: Modelling a multiplex brain network by local transfer entropy
Source: Sci Rep. 2021 Jul 30;11:15525. doi: 10.1038/s41598-021-93190-z (PMC8324877; doi:10.1038/s41598-021-93190-z)
Supplement: Supplementary file 1 — Supplementary Information. [file 41598_2021_93190_MOESM1_ESM.pdf]

Contents

1 Workflow of the analytical approach used in this paper. 3

2 Brain areas (ROIs) considered in this paper. 4

3 Statistical significance of dyadic interactions 5

4 Subgraphs in a multiplex network 7

5 Multireciprocity and synchronous or sequential mechanisms. 10

6 Quantitative study of Centrality indexes 12

7 Single links analysis 15

8 Bibliography. 19

Local Transfer Entropy in Brain Functional Modules. (SUPPLEMENTARY MATERIALS)

Fabrizio Parente* and Alfredo Colosimo*

*Dept. of Anatomy. Histology. Forensic Medicine and Orthopaedics Sapienza. University of Rome

1 Workflow of the analytical approach used in this paper.

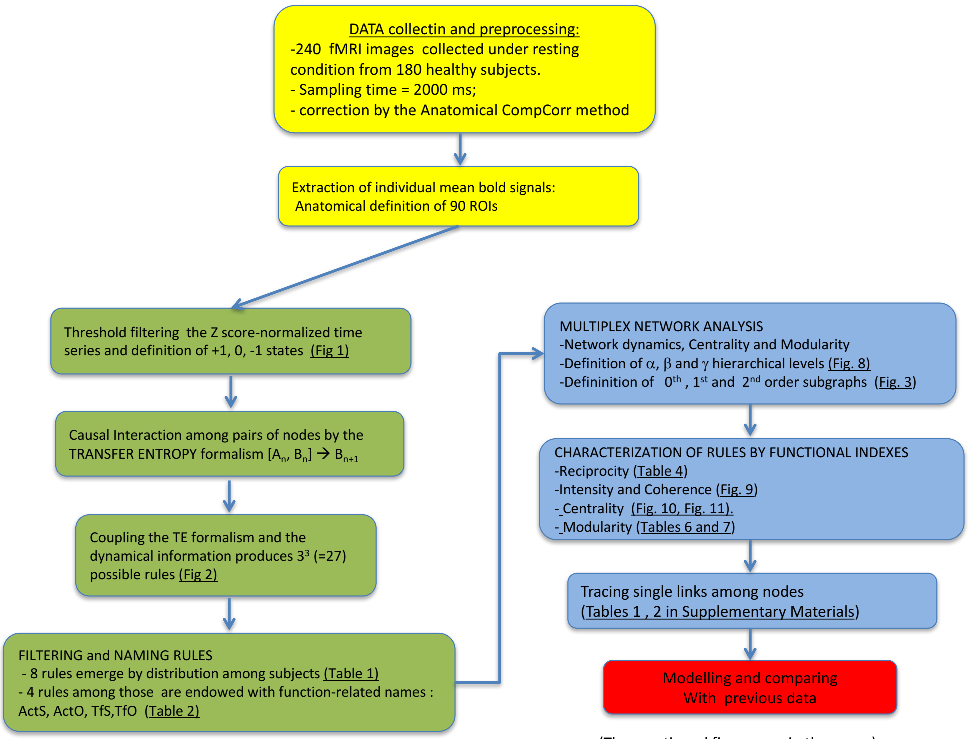

(The mentioned figures are in the paper).

Figure S1: *Flowchart of the analytical approach followed in this paper.*
- Yellow boxes = Preliminary steps (Section 2.1 of the paper): data collection and preprocessing and signal extraction by the defined 90 ROIs, .
- Green boxes = Characterizing the dyadic brain region interactions (Section 2.2 of the paper and Section 3 of Suppl. Mat., below): a) time-series discretization and characterization of the three functional states (1, 0 and -1); b) estimating the causal interaction by the Transfer Entropy (TE) formalism of a whole set of 27 state combinations (rules); c) finding a significant subset of function-related rules, namely ActS, TfS, ActO and TfO.
- Blue boxes = Multiplex network analysis (section 2.3 of the paper): a) characterizing the dynamics of the mutual interactions among ROIs from (90x89=8010, self-interaction excluded) adjacency matrices; b) studying the adjacency matrices associated to the four above rules by four network indexes, two of them to estimate dynamical features (reciprocity and subgraphs), and the other two for the centrality and modularity analysis; c) exploring the single links among ROIs in order to define possible functional circuits;
- Red Box = Comparative and critical final considerations (section 4.3.2 in this paper).

[Index]

2 Brain areas (ROIs) considered in this paper.

1	Central region		Precentral gyrus	PRE (L/R)
2			Postcentral gyrus	POST (L/R)
3			Rolandic operculum	RO (L/R)
4	Frontal lobe	Lateral surface	Superior frontal gyrus, dorsolateral	F1 (L/R)
5			Middle frontal gyrus	F2 (L/R)
6			Inferior frontal gyrus, opercular part	F3OP (L/R)
7			Inferior frontal gyrus, triangular part	F3T (L/R)
8		Medial surface	Superior frontal gyrus, medial	F1M (L/R)
9			Supplementary motor area	SMA (L/R)
10			Paracentral lobule	PCL (L/R)
11		Orbital surface	Superior frontal gyrus, orbital part	F1O (L/R)
12			Superior frontal gyrus, medial orbital	F1MO (L/R)
13			Middle frontal gyrus, orbital part	F2O (L/R)
14			Inferior frontal gyrus, orbital part	F3O (L/R)
15			Gyrus rectus	GR (L/R)
16			Olfactory cortex	OC (L/R)
17	Temporal lobe	Lateral surface	Superior temporal gyrus	T1 (L/R)
18			Heschl gyrus	HES (L/R)
19			Middle temporal gyrus	T2 (L/R)
20			Inferior temporal gyrus	T3 (L/R)
21	Parietal lobe	Lateral surface	Superior parietal gyrus	P1 (L/R)
22			Inferior parietal	P2 (L/R)
23			Angular gyrus	AG (L/R)
24			Supramarginal gyrus	SMG (L/R)
25		Medial surface	Precuneus	PQ (L/R)
26	Occipital lobe	Lateral surface	Superior occipital gyrus	O1 (L/R)
27			Middle occipital gyrus	O2 (L/R)
28			Inferior occipital gyrus	O3 (L/R)
29		Medial/Inferior surface	Cuneus	Q (L/R)
30			Calcarine fissure and surrounding cortex	V1 (L/R)
31			Lingual gyrus	LING (L/R)
32			Fusiform gyrus	FUSI (L/R)
33	Limbic lobe		Temporal pole: superior temporal gyrus	T1P (L/R)
34			Temporal pole: middle temporal gyrus	T2P (L/R)
35			Anterior cingulate and paracingulate gyri	ACIN (L/R)
36			Median cingulate and paracingulate gyri	MCIN (L/R)
37			Posterior cingulate gyrus	PCIN (L/R)
38			Hippocampus	HIP (L/R)
39			Parahippocampal gyrus	PHIP (L/R)
40	Insula		Insula	IN (L/R)
41	Sub cortical gray nuclei		Amygdala	AMYG (L/R)
42			Caudate nucleus	CAU (L/R)
43			Lenticular nucleus, putamen	PUT (L/R)
44			Lenticular nucleus, pallidum	PAL (L/R)
45			Thalamus	THA (L/R)

Figure S2: *Synthetic names of the 90 ROIs considered in this paper.*
The emispheric location of each ROI is indicated by L (Left) and R (Right).

[Index]

3 Statistical significance of dyadic interactions

Given the 90 brain regions [1] in **Figure S2**, in order to study the significant distribution among subjects of the 27 (3^3) rules from the dyadic interactions, for each subject a square matrix of 8010 local Transfer Entropy (TE) [2] values (excluding 90 self-interactions) was considered and the rules associated to random events were filtered out by a series of statistical tests. Thus, since only the time-sequences are randomized, the marginal frequency of events in each brain region is unchanged, and the local TE could be re-calculated for each brain region combination, for all subjects and rules.

At increasing signal threshold the significant events increase [3] [4], and the noise over the information transfer among brain regions is reduced, as confirmed by the Mean Square Error (MSE) between the local TE values of each rule for each subject and the corresponding random model. For some rules an increasing trend in both the sum of local TE and MSE points to a significant improvement of the information transfer at increasing signal threshold. Those rules should emerge as different from other rules at the highest signal threshold (= 1 S.D.), which is confirmed by a one way ANOVA (shown in the Results section of the main text).

Since MSE only accounts for symmetrical deviations from the random model, to identify rules with an increasing trend of TE values, a t-student for independent samples (Bonferroni corrected for the number of subjects) was performed. The rationale is that a significant decrease with respect to the random model indicates disadvantaged rules, in contrast with rules showing a significant increases of TE values, which are advantaged and possibly endowed with a functional meaning. In conclusion, we take as significant the rules characterized by the following properties:
- increasing trend of TE values in parallel with signal threshold;
- increasing trend of MSE in parallel with z-score threshold;
- significantly higher values of local TE when compared with other rules;
- significant probability in a right-tail statistical comparison test.

In **Figure S3** a concise scheme of the whole procedure is reported.

[Index]

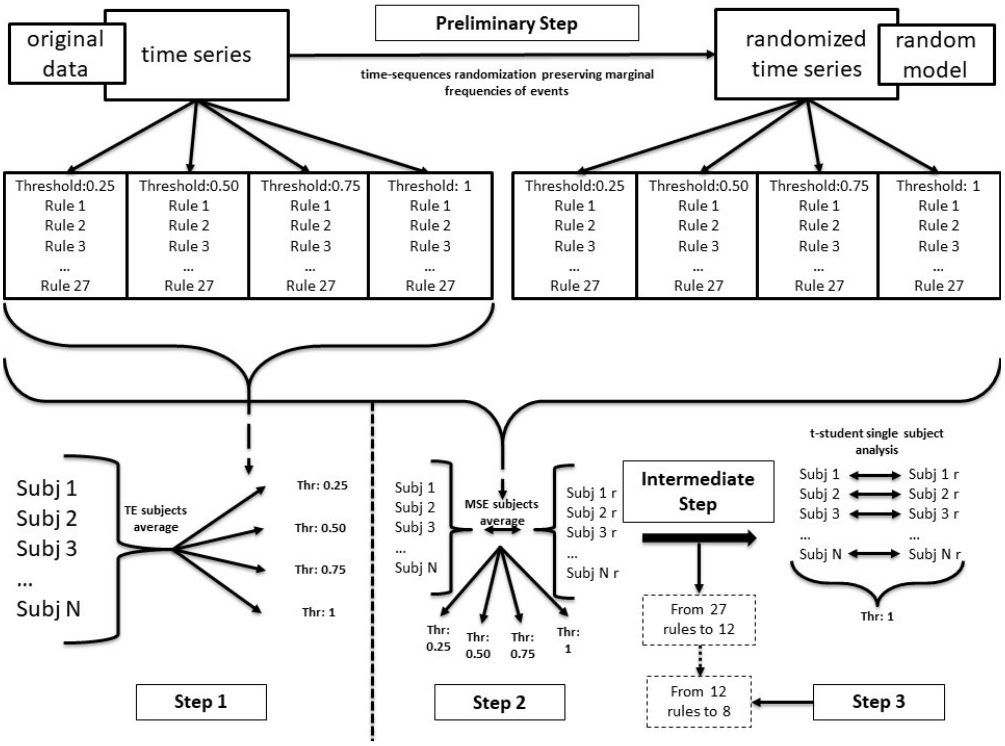

Figure S3: *Statistical significance of the rules.*
To evaluate the significance of rules the following steps were used:
- in the preliminary step (top center) the time-series at hand were randomized preserving the marginal frequencies of events (1, -1 and 0);
- in step 1 (bottom left) for each subject the TE average values for each rule (27) and for each threshold (0.25, 0.50, 0.75, 1) were calculated;
- in step 2 (bottom right) the average Mean Square Error (MSE) between original data and random model were calculated for all rules and thresholds;
- in the intermediate step only rules having an increasing trend of values (calculated in step 1 and 2) as a function of threshold were selected, that is 12 rules out of 27;
- in step 3 (bottom right) for each subject the original and randomized data at the highest threshold (1) were analyzed by a t test right-tail, in order to filter out rules having TE values higher than the random model.
- In the very last step 8 rules out of 12 showed significant results in the largest fraction of subjects.

[Index]

4 Subgraphs in a multiplex network

Different names are possible for a 3-nodes subgraph [5] [6] [7] due to its symmetric features, e.g. ActO/ActS/ActO = ActS/ActO/ActO = ActO/ActO/ActS. In any case, we use the standardized name shown in the left panel of **Figure S4** and **Figure S5**.

In a Cycle triad the information transfer starts and ends at the same node. Conversely, in a Flux triad the information transfer starts from a source node and ends to a sink node, passing through a middle node, and the process was called **feedforward loop** [8]. Thus, in a Flux subgraph we characterize:
- a source node with only out- connections;
- a sink node with only in- connections;
- a middle node with both in- and out- connections.

Introducing Flux subgraphs in a multiplex frame needs to distinguish links passing between:
- source and middle nodes;
- middle and sink nodes;
- source and sink nodes.

Thus, a Flux subgraph is identified by the layers having a link corresponding to the previously defined cases and in the same order.

For example, the 2nd order subgraph: Flux ActO/ActS/TfO is formed by:
1) a link between source and middle nodes falling in the ActO layer;
2) a link between middle and sink nodes belonging to the ActS layer, and
3) a link between source and sink nodes belonging to the TfO layer.

[Index]

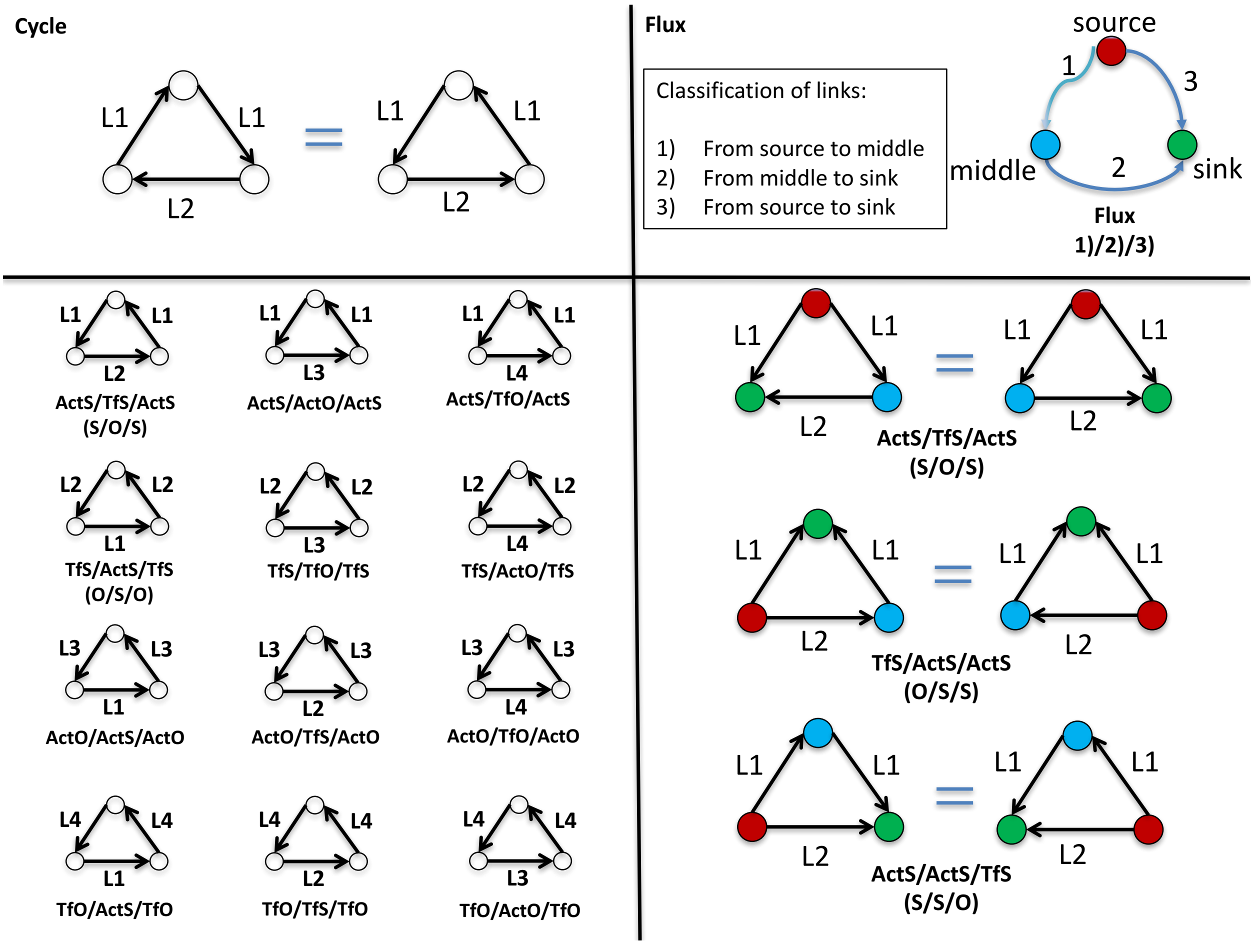

Figure S4: *Dynamic processes in* 1st *order triads (distributed in two layers):*
Left panel Top: isophorms of a Cycle, producing the same dynamical process; Bottom: names of the isophorms.
Right panel Top: information transfer pathways in a Flux triad. Bottom: Examples of 1st order Flow subgraphs. The combination of Flux subgraphs are named and characterized by the layers including, in the order, the 1st , 2nd and 3rd link.
In both panels the names of the corresponding β-hierarchical level are in parenthesis below the subgraphs.
[Index]

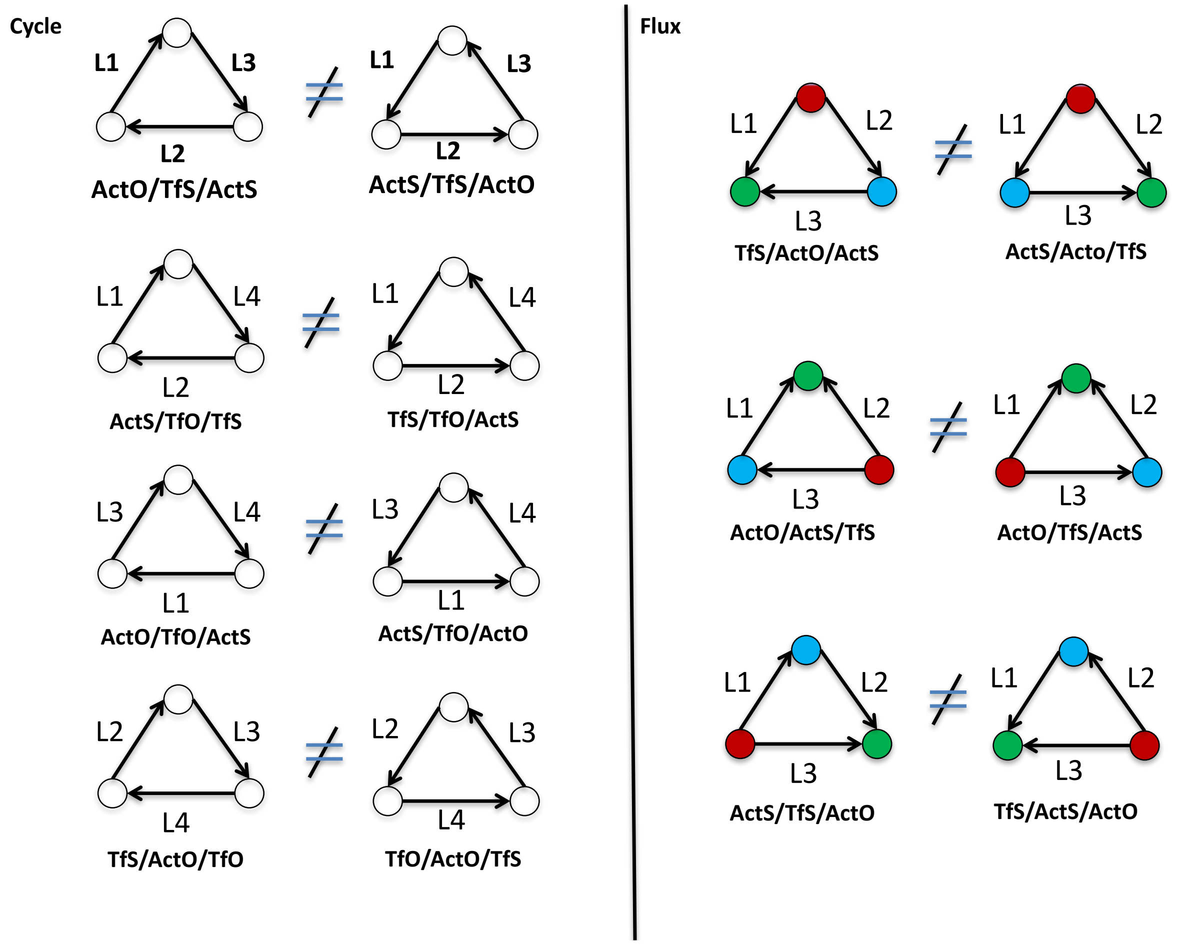

Figure S5: *Dynamic processes in* 2nd *order triads (distributed in three layers):*
Left panel: Triads in three interacting layers.
Right panel: Example of 2nd order Flux subgraphs, (only three layers (Acts, Tfs, ActO) out of four are considered).
Using three layers the symmetry found in the 1nd order subgraphs (**Figure S4**) is lost and different dynamic processes are included in the isophorms.
[Index]

5 Multireciprocity and synchronous or sequential mechanisms.

In order to characterize possible dynamical mechanisms underlying the network architecture, the logical functioning of the rules can be calculated between the interacting rules in their multireciprocal structure. Two kinds of dynamics can be assumed: synchronous and sequential. In the first case nodes can act in the same time step changing their state simultaneously. The only combination for such a case is the reciprocal turn off rules in their own layer: TfS/TfS and TfO/TfO. However, this combination is not-reciprocal in our results, and both Turn Off rules appear unidirectional. In the second case, node A could act on node B at time step n, and node B on node A at the next n+1 step, pointing to a temporal sequence changing the state of both A and B.

Results of the multireciprocity analysis show the following significant reciprocal interaction rules: TfS/TfO, ActS/TfO, ActO/TfS. As shown in the upper panel of **Figure S6**, the TfS/TfO rules cannot act at the same time: TfS need the node state of A=1 (or -1) and B=-1 (or 1), while the TfO the node state A=1 (of -1) and B=1 (or -1), then since they need different combination of nodes state to work, they cannot operate at the same time. On the other hand, in the turn off rules the node A change the state of the node B in 0, and vice versa, then the node B does not available to carry any reciprocal action. Consequently, TfS and TfO cannot describe a reciprocal dynamics, but operate independently reciprocally. In the latter cases, both ActS/TfO and ActO/TfS share same properties: they cannot operate synchronously, but a sequence dynamics is possible. As described in the bottom panel of **Figure S6**, if the node A acts the ActS (or ActO) rule on the node B, the state of node B change to 1 (or -1) state at the n+1 time step; then, the node B is now ready to operate the Turn Off rule to node A using the reciprocal connection. Such a dynamics can describe a feedback mechanism between nodes A and B, in which the first (de-)activates the second one, and the second turn off the first one sequencenly.

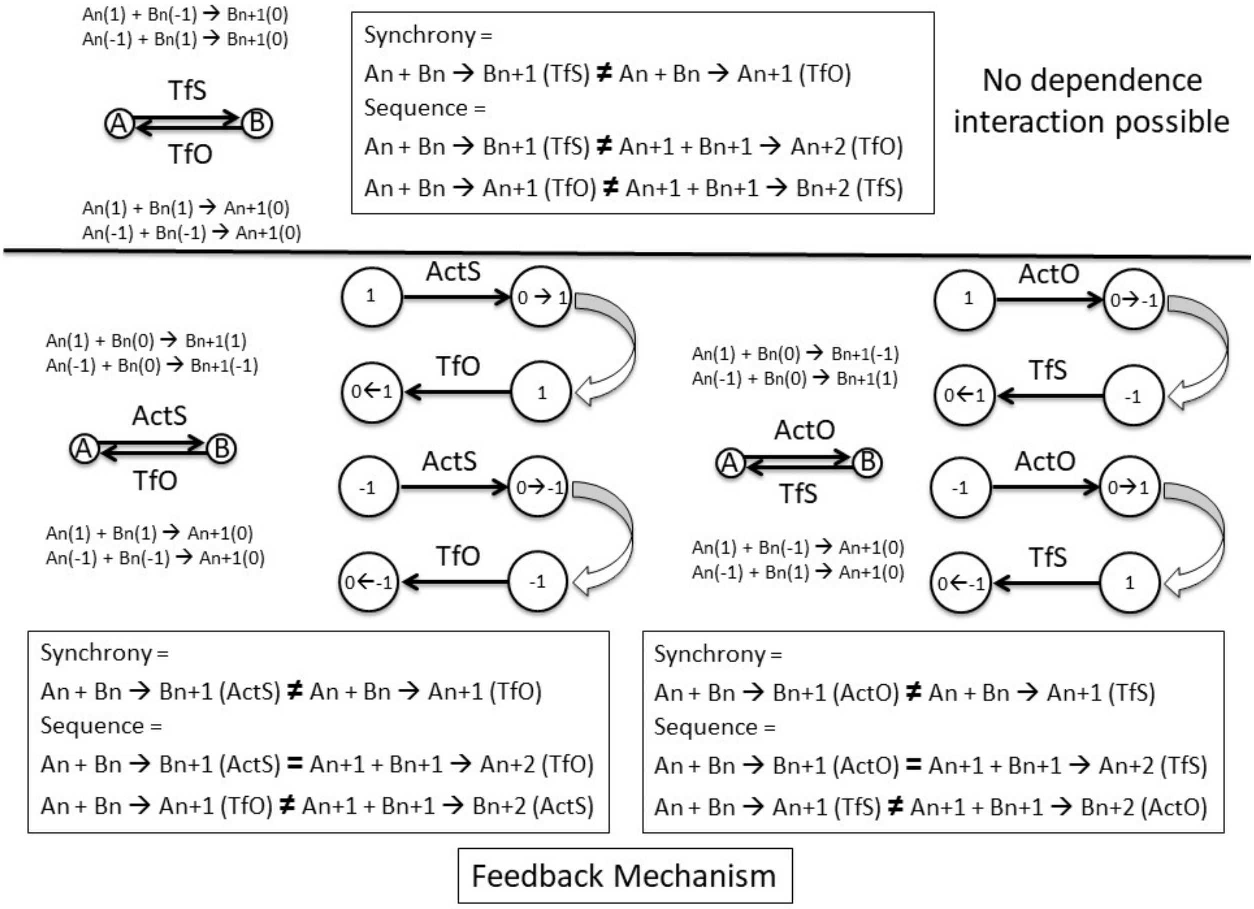

Figure S6: *Dynamical characterization of multireciprocity: reciprocal connection between different layers.* *Upper panel* Nodes acting with TfS and TfO tend to be reciprocal as shown in the left part of the panel. However, the dynamical characterization for both synchronous or sequential mechanisms is not satisfied . *Bottom panel* Nodes operating with ActS tend to be reciprocal turned off by the TfO rule (on the left), as well as nodes reciprocally connected by ActO and TfS (on the right). Both cases do not satisfy the synchronization mechanism, but can be related by a sequence dynamics in which: first, node B (de-)activates node A, then node A turns off node B in the following step. Such a dynamics describes a possible feed-back mechanism between reciprocal nodes, whose schematic representation is shown.

[Index]

6 Quantitative study of Centrality indexes

In the Centrality analysis [9] nodes can be sorted by specific features accounting for interactions within the whole system. The node degree is used to characterize node centrality and, in a directed and weighted network, the corresponding metrics. Thus, in-, out- and total-strength indicate nodes endowed with a major in-Flux or out-Flux. In particular, we defined as central the nodes having a value higher than, at least, the third quartile of its distribution ($>$75%). In a multilayer description, besides the study of the node degree distribution in each layer, the distribution of central nodes across different layers was reckoned according to the following definitions:
- Local central node: if present in not more than 25% of the total layers;
- Intermediate multiplex central nodes: if present in the range between 25% and 75% of the total layers;
- Multiplex central nodes: if present in more than 75% of total layers.

In **Figure S7** central nodes at the α-hierarchical level are shown: in the ActS rule many central nodes fall in the DMN/limbic and vision related brain regions, as in- and out-strength; the ActO central nodes belong to the fronto-parietal brain areas both as in- and out-strength; the TfS and TfO have both the basal ganglia as central nodes (in- for TfS and out- for TfO), while TfO and ActO share the fronto-parietal brain regions mainly as in-strength. Only intermediate multiplex central nodes (between two layers) are found:

- ActS and TfS $<-$ MCIN (in-), PHIP (out-) and T1 (out-); - ActO and TfO $<-$ F2 (out- and in-), F2O (in-), F3T (out-), P2, PCIN (out-) and PAL (out-) - TfS and TfO $<-$ F1O (in-), CAU out, PUT (out-), PAL (out-) and THA (out-).

In **Figure S8** the β- and γ-hierarchical level are considered: in the S rule nodes overlapping the pattern of the ActS rule appear central, while the basal ganglia are hidden; in the O rule both central nodes of ActO and TfO are detected; only the inferior frontal pars orbitalis (F3O) remains as intermediate multiplex central nodes between the two layers. Finally, in the γ-hierarchical level T matrix the whole pattern of central nodes are expressed: fronto-parietal, DMN/limbic and basal ganglia.

[Index]

Centrality

ActS In	ActS Out	ActS Tot
F1MO (L)* 0.72 (0.21)	F1MO (R) 0.69 (0.17)	F1MO (L)* 1.39 (0.22)
FIMO (R) 0.70 (0.21)	ACIN (R) 0.70 (0.17)	F1MO (R)* 1.39 (0.25)
MCIN (L)* 0.71 (0.18)	PHIP (L)** 0.73 (0.19)	PHIP (L) 1.37 (0.22)
MCIN (R)* 0.73 (0.20)	PHIP (R)** 0.74 (0.20)	PHIP (R)* 1.41 (0.21)
PCIN (L) 0.70 (0.23)	FUSI (L)* 0.72 (0.19)	Q (L) 1.37 (0.25)
PCIN (R)* 0.71 (0.23)	FUSI (R)** 0.74 (0.20)	LING (L) 1.36 (0.28)
HIP (R) 0.70 (0.20)	POST (R) 0.71 (0.26)	LING (R)* 1.40 (0.23)
V1 (L)* 0.73 (0.20)	T1 (L) 0.70 (0.17)	O2 (L) 1.36 (0.26)
V1 (R) 0.70 (0.19)	T1 (R)* 0.72 (0.18)	FUSI (L) 1.38 (0.27)
Q (L) 0.69 (0.19)		FUSI (R)** 1.43 (0.24)
LING (L) 0.70 (0.19)		POST (R) 1.35 (0.27)
LING (R) ** 0.74 (0.23)		T1 (L)* 1.41 (0.23)
O2 (L)* 0.71 (0.21)		T1(R) 1.38 (0.25)
O3 (R) 0.69 (0.22)		T1P (R) * 1.42 (0.27)
FUSI (R) 0.69 (0.21)		
T1 (L) * 0.71 (0.19)		
T1P (L) 0.70 (0.18)		
T1P (R) ** 0.74 (0.21)		

TfS In	TfS Out	TfS Tot
F10 (L) 0.31 (0.09)	F20 (L)* 0.31 (0.08)	OC (L) 0.61 (0.10)
SMA (R)* 0.32 (0.09)	F3O (L) 0.30 (0.07)	AMYG (L)** 0.64 (0.08)
OC (L) 0.31 (0.09)	F30 (R)** 0.32 (0.07)	AMYG (R) 0.61 (0.09)
MCIN (L) 0.31 (0.08)	PHIP (L)* 0.31 (0.06)	CAU (L)** 0.64 (0.10)
MCIN (R) 0.31 (0.08)	AMYG (L)** 0.32 (0.07)	CAU (R)** 0.64 (0.10)
AMYG (L)* 0.32 (0.08)	AMYG (R) 0.30 (0.08)	PUT (L)* 0.62 (0.10)
AMYG (R) 0.31 (0.07)	PCL (L)* 0.31 (0.08)	PAL (L) 0.61 (0.09)
CAU (L)** 0.33 (0.09)	CAU (L)* 0.31 (0.08)	THA (L)* 0.63 (0.10)
CAU (R)** 0.33 (0.09)	CAU (R) 0.30 (0.08)	THA (R) 0.61 (0.11)
PUT (L)* 0.32 (0.09)	PUT (L) 0.30 (0.08)	T3 (L)* 0.62 (0.10)
PUT (R) 0.31 (0.08)	PAL (L)* 0.31 (0.08)	
	THA (L)** 0.32 (0.08)	
	THA (R)* 0.31 (0.08)	
	HES (L)* 0.31 (0.08)	
	HES (R)* 0.31 (0.07)	
	T1(R)* 0.31 (0.07)	
	T1P (L)* 0.31 (0.07)	
	T1P (R) 0.30 (0.06)	
	T2 (L) 0.30 (0.07)	
	T2P (R) 0.30 (0.08)	
	T3 (L)** 0.32 (0.07)	
	T3 (R)** 0.32 (0.07)	

ActO In	ActO Out	ActO Tot
F1 (R) 0.52 (0.18)	F2 (L)** 0.54 (0.16)	F2 (L)* 1.05 (0.19)
F2 (L) 0.51 (0.16)	F2(R) 0.51 (0.15)	F2 (R)** 1.07 (0.21)
F2 (R)** 0.56 (0.19)	F3OP (L)** 0.54 (0.17)	F2O (L) 1.00 (0.23)
F2O (L) 0.52 (0.18)	F3OP (R)** 0.54 (0.18)	F2O (R)* 1.04 (0.25)
F2O (R)** 0.57 (0.21)	F3T (L) 0.51 (0.17)	F3OP (L)** 1.06 (0.23)
F3OP (L) 0.52 (0.17)	SMA (L)* 0.53 (0.16)	F3OP (R)** 1.06 (0.24)
F3OP (R) 0.52 (0.18)	SMA(R)** 0.55 (0.19)	F3T (L)* 1.03 (0.23)
F3T (L) 0.52 (0.18)	IN (L)* 0.53 (0.16)	F3T (R) 1.00 (0.21)
F3T (R)* 0.53 (0.18)	IN (R) 0.50 (0.17)	P2 (L) 1.01 (0.20)
P2 (R) 0.52 (0.18)	PCIN (R) 0.50 (0.16)	P2 (R)* 1.04 (0.21)
AG (R) 0.51 (0.17)	P2 (L) 0.51 (0.15)	SMG (R) 1.00 (0.21)
	P2 (R)* 0.52 (0.15)	AG (R) 1.00 (0.18)
	SMG (R) 0.50 (0.15)	
	PUT (R) 0.50 (0.20)	
	PAL (R) 0.51 (0.18)	

TfO In	TfO Out	TfO Tot
F1O (L) 0.31 (0.08)	F2 (L) 0.32 (0.08)	F1 (L) 0.61 (0.09)
F1O (R)* 0.32 (0.09)	MCIN (R) 0.32 (0.09)	F1O (R) 0.61 (0.10)
F2 (R) 0.31 (0.10)	PCIN (R) 0.32 (0.08)	F2 (L) 0.61 (0.10)
F2O (R) 0.31 (0.08)	CAU (L)* 0.33 (0.09)	F2 (R)* 0.62 (0.10)
F3T (L) 0.31 (0.09)	CAU (R)** 0.34 (0.09)	F2O (L) 0.61 (0.10)
F3T (R)** 0.33 (0.09)	PUT (L)* 0.33 (0.09)	F2O (R) 0.61 (0.09)
F3O (R) 0.31 (0.09)	PAL (L)* 0.33 (0.08)	SMA (L) 0.61 (0.09)
GR (L) 0.31 (0.09)	PAL (R) 0.32 (0.08)	P2 (L) 0.60 (0.08)
GR (R) 0.31 (0.08)	THA (L) 0.32 (0.09)	CAU (L) 0.61 (0.11)
P2 (L) 0.31 (0.08)	THA (R) 0.32 (0.08)	CAU (R)* 0.62 (0.10)
P2 (R) 0.31 (0.09)		PUT (L)** 0.63 (0.10)
AG (R) 0.31 (0.08)		PAL (L)* 0.62 (0.11)
		PAL(R)* 0.62 (0.09)
		THA (L) 0.61 (0.10)
		THA (R) 0.61 (0.10)

Figure S7: *Centrality Index of ActS, TfS, ActO and TfO Rules.* Different rules show a different pattern of central nodes, indicating a possible different functional role characterizing each cerebral brain region. Central regions are defined as having values higher than the third quartile of the distribution. The asterisk (*) indicates values higher than 85% of the distribution and double asterisk (**) higher than 95%.

[Index]

Centrality								
S			O			T		
IN	OUT	TOT	IN	OUT	TOT	IN	OUT	TOT
F1MO (L) 1.00 ± 0.23	F3O (L) 0.98 ± 0.22	F1MO (L) 1.95 ± 0.21	F1 (R) 0.82 ± 0.22	F2 (L)** 0.86 ± 0.20	F2 (L)* 1.66 ± 0.23	F1MO (L)** 1.75 ± 0.26	PRE (R) 1.71 ± 0.21	SMA (R) ** 3.41 ± 0.31
MCIN (L)* 1.03 ± 0.23	F3O (R) 1.00 ± 0.23	F1MO (R) 1.95 ± 0.24	F1O (R) 0.81 ± 0.19	F2 (R) * 0.82 ± 0.20	F2 (R)** 1.69 ± 0.22	F1MO (R) 1.70 ± 0.23	F3O (L) 1.71 ± 0.18	F1MO (L) * 3.38 ± 0.31
MCIN (R)** 1.04 ± 0.23	ACIN (R) 0.98 ± 0.19	PHIPP (L)* 1.97 ± 0.24	F2 (L) 0.80 ± 0.20	F3O (L) * 0.83 ± 0.21	F2O (L) 1.61 ± 0.26	GR (L) 1.71 ± 0.28	RO (L) 1.71 ± 0.19	F1MO (R) 3.37 ± 0.31
PCIN (L) 0.99 ± 0.24	PHIP (L)** 1.04 ± 0.17	PHIPP (R)* 1.98 ± 0.23	F2 (R) ** 0.87±0.22	F3O (R) * 0.83 ± 0.21	F2O (R)* 1.65 ± 0.27	PCIN (P) ** 1.75 ± 0.26	SMA (L) 1.73 ± 0.20	V1 (L) 3.37 ± 0.29
PCIN (R) 1.01 ± 0.27	PHIP (L)** 1.04 ± 0.15	LING (R)* 1.97 ± 0.27	F2O (L) 0.83 ± 0.19	SMA (L)* 0.83 ± 0.20	F3OP (L) * 1.65 ± 0.26	V1 (L) * 1.74 ± 0.21	SMA (R)** 1.77 ± 0.21	V1 (R) 0.37 ± 0.29
HIP (R) 1.00 ± 0.21	FUSI (L)* 1.01 ± 0.19	FUSI (L) 1.96 ± 0.27	F2O (R)** 0.88 ± 0.24	SMA (R)* 0.84 ± 0.22	F3OP (R)* 1.65 ± 0.26	V1 (R) 1.71 ± 0.21	IN (L) 1.72 ± 0.17	LING (R)* 3.39 ± 0.30
V1 (L)* 1.02 ± 0.23	FUSI (R)* 1.03 ± 0.22	FUSI (R)** 2.00 ± 0.25	F3O (L) 0.82 ± 0.19	IN (L) 0.81 ± 0.19	F3T (L) 1.61 ± 0.24	LING (R) * 1.72 ± 0.24	PUT (L) 1.71 ± 0.20	T1 (L) 3.38 ± 0.27
LING (L): 0.99 ± 0.21	POST (R): 0.98 ± 0.28	T1 (L)* 1.98 ± 0.24	F3O (R) 0.82 ± 0.21	PCIN (L) 0.80 ± 0.20	F3T (L) 1.60 ± 0.23	CAU (L) 1.72 ± 0.26	PUT (R) 1.72 ± 0.23	
LING (R) ** 1.04 ± 0.26	T1 (L) 0.98 ± 0.21	T1 (R)* 1.97 ± 0.26	F3T (L) 0.83 ± 0.20	PCIN (R)* 0.82 ± 0.21	P2 (L) 1.61 ± 0.22	T1 (L)* 1.72 ± 0.20		
O2 (L) 0.98 ± 0.23	T1 (R)* 1.03 ± 0.21	T1P (R)** 2.02 ± 0.28	F3T (R)* 0.86 ± 0.21	P2 (L) 0.81 ± 0.20	P2 (R)* 1.64 ± 0.21	T1P (R) * 1.74 ± 0.23		
CAU (L) 0.98 ± 0.25	T2P (R) 0.98 ± 0.20		P2 (L) * 0.80 ± 0.19	P2 (R) 0.81 ± 0.19	AG (R) 1.58 ± 0.21			
T1 (L) 1.00 ± 0.22			P2 (R) 0.83 ± 0.20	CAU (L) 0.80 ± 0.18	PAL (R) 1.59 ± 0.23			
T1P (L) 0.99 ± 0.20			SMG (L) 0.81 ± 0.23	CAU (R) 0.83 ± 0.21				
T1P (R)** 1.04 ± 0.22			AG (R) 0.82 ± 0.20	PUT (L) 0.81 ± 0.21				
				PUT (R) 0.81 ± 0.25				
				PAL (L) * 0.82 ± 0.19				
				PAL (R) 0.83 ± 0.21				
				THA (L) 0.81 ± 0.22				
				THA (R) 0.80 ± 0.21				

Figure S8: *Centrality Index of β and γ hierarchical levels.* Higher hierarchical levels show similar pattern of central nodes as in the α-hierarchical level. The asterisk (*) indicates values higher than 85% of the distribution and double asterisk (**) higher than 95%.

[Index]

7 Single links analysis

Tracing single connections points to describe the possible regulation pathways of the brain network. For the four layers of the α-hierarchical level the highest 40 links (corresponding to the 0.5% of the total) are identified and reported in **Table S1** and **Table S2** .

In the left side of **Table 1**, 26 ActS links (65% of the total) concern the same contralateral brain region: among these, eight are reciprocal and ten are not reciprocal with a trend in the left to right lateralization (7). The remaining links tend to fall in the same functional or anatomical module:
- a) lateral frontal cortex (F3OP to F3T);
- b) medial frontal cortex (ACIN and GR to F1M);
- c) limbic (PHIP to HIP);
- d) visual cortex (V1 to LING, O2 to O1, O3 and FUSI to O2, FUSI to O3);
- e) temporal lobe (HES to T1, T1 to T1P);
- f) non-specific pathways (HES to RO).

Finally, most of the previous links (11/14) are homolateral (5/11 and 6/11 for right and left, respectively).

In summary, the ActS rule shows: 1) a connection to the same contralateral brain region mutual and non-mutual; 2) mutual bidirectional and non-mutual connections with non-homologous contralateral brain regions. Although some bidirectional connections were detected, the reciprocity analysis did not find any significant reciprocal connection. However, the ActS remains the only rule having a small trend in this direction, to be possibly ascribed to the cohesive nature of the rule. The non-homologous brain regions fall mainly in the same functional or structural network, e.g.: a) DMN with anterior cingulum to frontal medial/medial orbitalis, precuneus to posterior cingulum; b) cingulum cortex with anterior to middle cingulum; c) limbic with parahippocampus to hippocampus; d) vision with fusiform to calcarine, lingual and middle occipital gyrus. Thus, the above connections point to a dynamics falling within some specific functional modules of the brain regions.

The TfS links (**Table S1**, right side) enlighten the different pathways associated to central nodes: the basal ganglia, acting as out-strength nodes, take connections from some regions of cognitive and motor associated areas (PRE, SMA, RO, IN and T1), mainly towards the putamen (PUT). Thalamus and caudate point to other sparse regions: THA to F1M, PCIN and PQ; CAU to F2. Consequently, the same basal ganglia, acting as in-strength nodes, taking connection from:
- limbic and DMN (OC, GR, F1M, F1MO, ACIN) to the caudate (CAU),
- the lateral frontal (F2) and cingulate cortex (ACIN and MCIN) to the putamen (PUT).

Other connections include:
- temporo-frontal areas (F3O to T3),
- limbic and DMN (PHIP to F1M and F1M to AMYG),
- insula cortex (IN) to lateral frontal areas (F3O and F3OP), - occipital/visual cortex and DMN (Q to F1M, FUSI to PCIN, T3P to O3),
- the interacion between a sparse set of brain regions (T3 to HIP, F1 to PCL).

Thus, the basal ganglia seem to be an intermediate station starting from the limbic and the DMN towards the salience and motor network.

As for the ActO rule, a particular connectivity pattern arises (**Table S2, left side)**, since both the main out- and in-strength nodes belong to the lateral frontal and parietal

cortex. In particular:
- different regions of the DMN (F1MO, PCIN) are reached by connections coming from the F3O,
- the HIP is reached by the F2,
- the MCIN and the PQ by the F3T.
Same sources are detected for limbic and DMN areas taking connections from both motor and attention-related brain areas (SMA, SMG, P2 and IN). Conversely, DMN (F1MO, PQ) give projections towards the frontal regions (F2, F3OP and F3T).

Finally, regarding the TfO rule (**Table S2, right side)**:
- from a sparse set of brain areas belonging to temporal (T1, T2 and T3), occipital/visual (O1, FUSI and LING) and limbic/DMN (AMYG, PQ, PCIN) start many connections to the lateral part of the frontal lobe (F1O, F2, F2O, F3T and F3OP)
- the salience/motor network (PRE, SMA, MCIN, IN) and basal ganglia (PUT and PAL) take connections with the medial part of the lateral lobe (F1M, GR) and other libic/DMN brain regions (HIP, PCIN, AG, PQ).

[Index]

**ActS**			**TfS**		
Brain Regions	*Freq.*	*Mean(S.D.)*	*Brain Regions*	*Freq.*	*Mean(S.D.)*
F3OP (L) –> F3T (L)	0.96:	0.026 (0.016)	**PUT (L)** –> PRE (L)	0.83:	0.007 (0.005)
F3OP (R) –> F3T (R)	0.96:	0.027 (0.018)	**PUT (L)** –> PRE (R)	0.78:	0.007 (0.006)
F3O (L) –> F3O (R)	0.96:	0.018 (0.013)	**CAU (R)** –> F2 (R)	0.78:	0.007 (0.005)
RO (R) –> RO (L)	0.96:	0.025 (0.017)	IN (R) –> F3T (L)	0.82:	0.006 (0.004)
RO (L) –> RO (R)	0.98:	0.025 (0.017)	IN (L) –> F3O (R)	0.81:	0.006 (0.005)
HES (L) –> RO (R)	0.96:	0.019 (0.015)	**PUT (L)** –> RO (L)	0.78:	0.008 (0.006)
OC (R) –> OC (L)	0.97:	0.025 (0.017)	PUT (R) –> RO (L)	0.79:	0.006 (0.005)
**ACIN (R)** –> F1M (R)	1:	0.023 (0.017)	**PUT (L)** –> RO (R)	0.81:	0.008 (0.006)
**F1MO (R)** –> **F1MO (L) ***	0.99:	0.029 (0.018)	PUT (R) –> RO (R)	0.81:	0.007 (0.005)
GR (L) –> **F1MO (L) ***	0.96:	0.023 (0.016)	**PUT (L)** –> **SMA (R)**	0.79:	0.008 (0.005)
F1MO (L) –> **F1MO (R)**	0.96:	0.023 (0.015)	O1 (R) –> **OC (L)**	0.80:	0.007 (0.005)
IN (L) –> IN (R)	0.97:	0.026 (0.015)	**PHIP (L)** –> F1M (L)	0.80:	0.006 (0.006)
**ACIN (R)** –> ACIN (L)	0.99:	0.034 (0.018)	Q (L) –> F1M (L)	0.79:	0.006 (0.005)
ACIN (L) –> ACIN (R)	0.97:	0.019 (0.013)	**THA (L) **** –> F1M (L)	0.79:	0.007 (0.006)
MCIN (L) –> **MCIN (R) ***	0.98:	0.028 (0.017)	**PUT (L)** –> IN (R)	0.78:	0.006 (0.005)
PCIN(L) –> **PCIN (R) ***	0.96:	0.028 (0.016)	**PAL (L)** –> IN (R)	0.83:	0.006 (0.004)
**PHIP (L) **** –> HIP (L)	0.96:	0.022 (0.014)	FUSI (L) –> PCIN (L)	0.78:	0.007 (0.006)
**PHIP (L) **** –> **HIP (R)**	0.98:	0.019 (0.015)	FUSI (R) –> PCIN (L)	0.78:	0.006 (0.005)
**PHIP (R) **** –> PHIP (L)	0.97:	0.023 (0.015)	FUSI (L) –> PCIN (R)	0.79:	0.007 (0.005)
**PHIP (L) **** –> PHIP (R)	0.98:	0.024 (0.017)	**THA (L) **** –> PCIN (R)	0.78:	0.007 (0.005)
V1 (R) –> **V1 (L) ***	1:	0.031 (0.019)	**T3 (L) **** –> HIP (L)	0.80:	0.006 (0.005)
V1 (L) –> **V1 (R)**	0.97:	0.023 (0.016)	F1MO (L) –> **AMYG (L) ***	0.78:	0.007 (0.005)
Q (L) –> Q (R)	0.99:	0.025 (0.016)	T3P (R) –> O3 (R)	0.78:	0.006 (0.004)
LING (R) –> **LING (L)**	0.98:	0.023 (0.016)	**THA (L) **** –> PQ (L)	0.80:	0.006 (0.005)
V1 (R) –> **LING (R) ****	0.97:	0.026 (0.017)	F1 (L) –> PCL (L)	0.82:	0.006 (0.004)
LING (L) –> **LING (R) ****	0.99:	0.027 (0.016)	F1M (L) –> **CAU (L) ***	0.81:	0.007 (0.005)
O1 (R) –> O1 (L)	0.97:	0.021 (0.015)	F1MO (L) –> **CAU (L) ***	0.80:	0.007 (0.006)
O2 (R) –> O1 (R)	0.96:	0.019 (0.014)	F1MO (R) –> **CAU (L) ***	0.80:	0.008 (0.006)
O2 (R) –> **O2 (L)**	0.97:	0.024 (0.015)	GR (L) –> **CAU (L) ***	0.83:	0.008 (0.006)
O3 (L) –> **O2 (L)**	0.96:	0.019 (0.015)	ACIN (L) –> **CAU (L) ***	0.80:	0.007 (0.005)
**FUSI (L) *** –> **O2 (L)**	0.96:	0.024 (0.016)	OC (R) –> **CAU (R) ****	0.78:	0.007 (0.005)
**FUSI (R) **** –> O3 (R)	0.96:	0.026 (0.016)	F1MO (R) –> **CAU (R) ****	0.79:	0.006 (0.004)
POST (L) –> POST (R)	0.98:	0.022 (0.014)	ACIN (R) –> **CAU (R) ****	0.78:	0.007 (0.005)
SMG (R) –> SMG (L)	0.98:	0.022 (0.014)	PAL (R) –> **CAU (R) ****	0.80:	0.006 (0.004)
SMG (L) –> SMG (R)	0.99:	0.026 (0.017)	F2 (L) –> **PUT (L)**	0.78:	0.005 (0.004)
PQ (L) –> PQ (R)	0.99:	0.033 (0.019)	ACIN (L) –> **PUT (L)**	0.79:	0.007 (0.005)
PUT (R) –> PUT (L)	0.97:	0.023 (0.015)	MCIN (L) –> **PUT (R)**	0.79:	0.005 (0.004)
PUT (L) –> PUT (R)	0.97:	0.023 (0.014)	**PUT (L)** –> T1 (L)	0.80:	0.007 (0.005)
HES (R) –> **T1 (L) ***	0.97:	0.022 (0.015)	PUT (R) –> T1 (L)	0.80:	0.006 (0.0059
**T1 (R) *** –> **T1P (R) ****	0.97:	0.023 (0.015)	**F3O (R) **** –> T3 (L)	0.79:	0.005 (0.004)

Table S1: *ActS and TfS single links.*
1) The ActS rule shows mutual interactions between interhemispheric (right and left) part of the same cerebral region, intrahemispheric interactions between different brain regions, and recursive pathway within the same functional module;
2) the TfS rule describes a basal ganglia (caudate and putamen) pathway toward frontal and DMN/limbic brain regions.
Minimal fraction (0.5% = 40 connections) of the highest value weight links.
In bold central nodes of in- and out-strength.
Asterisk (*) and double asterisk (**) indicate a strength of the distribution values higher than 85% and 95%, respectively.

**ActO**			**TfO**		
Brain Regions	*Freq.*	*Mean(S.D.)*	*Brain Regions*	*Freq.*	*Mean(S.D.)*
PRE (L) –> **F1 (R)**	0.83:	0.013 (0.010)	POST (R) –> F1 (R)	0.81:	0.006 (0.005)
F1MO (R) –> **F2 (L)**	0.84:	0.011 (0.010)	AMYG (L) –> F1O (R)	0.78:	0.006 (0.004)
F1MO (L) –> **F2 (R) ** **	0.90:	0.016 (0.012)	FUSI (L) –> F2 (L)	0.79:	0.006 (0.005)
F1MO (R) –> **F3OP (L)**	0.87:	0.012 (0.010)	O1 (R) –> **F2 (R)**	0.77:	0.006 (0.005)
PQ (L) –> **F3OP (L)**	0.84:	0.013 (0.011)	POST (R) –> **F2 (R)**	0.77:	0.007 (0.005)
F1MO (L) –> **F3OP (R)**	0.83:	0.012 (0.011)	T2 (L) –> **F2 (R)**	0.76:	0.006 (0.005)
F1MO (R) –> **F3OP (R)**	0.83:	0.012 (0.010)	T3 (R) –> **F2 (R)**	0.76:	0.009 (0.007)
F1MO (R) –> **F3T (L)**	0.83:	0.014 (0.009)	FUSI (L) –> F2O (L)	0.76:	0.006 (0.005)
PQ (L) –> **F3T (L)**	0.84:	0.012 (0.009)	T1 (L) –> **F2O (R)**	0.76:	0.006 (0.005)
F1MO (L) –> **F3T (R) ***	0.85:	0.014 (0.011)	T2 (L) –> F3OP (R)	0.77:	0.007 (0.006)
PRE (R) –> F1M (L)	0.86:	0.012 (0.009)	PQ (L) –> **F3T (L)**	0.77:	0.006 (0.004)
PRE (R) –> F1M (R)	0.83:	0.013 (0.011)	LING (L) –> **F3T (R) ****	0.76:	0.006 (0.004)
**PUT (R)** –> F1M (R)	0.83:	0.013 (0.010)	F1 (L) –> **F3O (R)**	0.76:	0.009 (0.007)
**F3OP (R) **** –> F1MO (L)	0.88:	0.018 (0.014)	**PCIN (R)** –> **F3O (R)**	0.78:	0.006 (0.004)
**IN (L) *** –> F1MO (L)	0.85:	0.018 (0.015)	P2 (L) –> SMA (R)	0.77:	0.008 (0.005)
**IN (R)** –> F1MO (L)	0.86:	0.018 (0.013)	PAL (L) –> OC (L)	0.76:	0.006 (0.005)
**SMG (R)** –> F1MO (L)	0.85:	0.014 (0.011)	PRE (L) –> F1M (L)	0.78:	0.007 (0.005)
PRE (L) –> F1MO (R)	0.85:	0.011 (0.010)	PRE (R) –> F1M (L)	0.78:	0.008 (0.005)
**SMA (L) *** –> F1MO (R)	0.85:	0.015 (0.012)	SMA (R) –> F1M (L)	0.78:	0.006 (0.004)
**SMA (R) **** –> F1MO (R)	0.83:	0.017 (0.014)	PRE (R) –> F1M (R)	0.78:	0.007 (0.005)
**IN (L) *** –> GR (L)	0.88:	0.016 (0.013)	SMA (L) –> **GR (L)**	0.80:	0.007 (0.005)
**IN (R)** –> GR (L)	0.84:	0.014 (0.011)	**PUT (L) *** –> **GR (L)**	0.78:	0.007 (0.005)
F3T (L) –> MCIN (R)	0.84:	0.014 (0.011)	IN (L) –> **GR (R)**	0.80:	0.006 (0.005)
PRE (L) –> PCIN (R)	0.92:	0.014 (0.010)	**MCIN (R)** –> **GR (R)**	0.77:	0.007 (0.005)
**F3OP (L) **** –> PCIN (R)	0.92:	0.016 (0.013)	**PUT (L) *** –> **GR (R)**	0.80:	0.007 (0.005)
**F3OP (R) **** –> PCIN (R)	0.83:	0.015 (0.011)	F3OP (R) –> PCIN (L)	0.80:	0.006 (0.005)
**SMA (L) *** –> PCIN (R)	0.83:	0.019 (0.014)	SMA (L) –> PCIN (L)	0.78:	0.008 (0.006)
**F2 (R)** –> HIP (L)	0.86:	0.017 (0.012)	F3OP (R) –> PCIN (R)	0.80:	0.007 (0.005)
**F2 (L) **** –> HIP (R)	0.86:	0.014 (0.012)	SMA (R) –> PCIN (R)	0.77:	0.006 (0.005)
**P2 (R) *** –> LING (R)	0.87:	0.010 (0.008)	**PUT (L) *** –> PCIN (R)	0.77:	0.007 (0.005)
F1M (R) –> P1 (L)	0.85:	0.012 (0.010)	PUT (R) –> PCIN (R)	0.81:	0.007 (0.005)
ACIN (R) –> P1 (L)	0.86:	0.014 (0.011)	SMG (R) –> HIP (L)	0.78:	0.007 (0.005)
F1MO (L) –> SMG (R)	0.89:	0.012 (0.010)	F3OP (R) –> LING (R)	0.80:	0.006 (0.005)
AG (L) –> SMG (R)	0.85:	0.013 (0.011)	F3O (L) –> P1 (R)	0.77:	0.007 (0.004)
PUT (L) –> **AG (R)**	0.84:	0.013 (0.011)	Q (L) –> SMG (L)	0.77:	0.007 (0.005)
**PUT (R)** –> **AG (R)**	0.85:	0.014 (0.012)	PUT (R) –> AG (R)	0.84:	0.007 (0.005)
**SMA (L) *** –> PQ (L)	0.84:	0.012 (0.009)	PUT (R) –> PQ (R)	0.78:	0.007 (0.005)
F3T (L) –> PQ (R)	0.88:	0.016 (0.012)	P1 (L) –> PAL (R)	0.77:	0.006 (0.005)
**SMA (L) *** –> PQ (R)	0.89:	0.015 (0.010)	PQ (R) –> T1 (L)	0.77:	0.005 (0.005)
PUT (L) –> T2P (R)	0.84:	0.012 (0.009)	F2 (L) –> T1 (R)	0.80:	0.006 (0.005)

Table S2: *ActO and TfO single links.*
1) The ActO rule shows a recursive interactions between the DMN/libic and fronto-parietal brain regions; 2) in TfO rule several pathways are detected: from a sparse set of cortical and sub-cortical brain areas to the fronto-parietal and DMN/limbic brain regions.
Minimal fraction (0.5% = 40 connections) of the highest value weight links.
In bold central nodes of in- and out-strength.
Asterisk (*) and double asterisk (**) indicate a strength of the distribution values higher than 85% and 95%, respectively.

8 Bibliography.

References

[1] Tzourio-Mazoyer N., Landeau B., Papathanassiou D., Crivello F., Etard O., Delcroix N., Mazoyer B., and Joliot M. Automated anatomical labeling of activations in spm using a macroscopic anatomical parcellation of the mni mri single-subject brain. *Neuroimage*, 15(1):273–89, 2002. 5

[2] Vicente R., Wibral M., Lindner M., and Pipa G. Transfer entropy–a model-free measure of effective connectivity for the neurosciences. *J Comput Neurosci.*, 30(1):45–67, 2010. 5

[3] Parente F. and Colosimo A. Brain activity patterns under resting state. *Biophysics and Bioengineering Letters.*, 9:1–13, 2017. 5

[4] Tagliazucchi E., Balenzuela P., Fraiman D., and Chialvo D.R. Criticality in large-scale brain fmri dynamics unveiled by a novel point process analysis. *Front Physiol.*, 3:15, 2012. 5

[5] Dimitrova T., Petrovski K., and Kocarev L. Graphlets in multiplex networks. *Sci Rep.*, 10(1):1928, 2020. 7

[6] Onnela J.P., Saramäki J., Kertész J., and Kaski K. Intensity and coherence of motifs in weighted complex networks. *Physical Review E*, 71:065103, 2005. 7

[7] Takaguchi T. and Yoshida Y. Cycle and flow trusses in directed networks. *R Soc Open Sci.*, 3(11):160270, 2016. 7

[8] Milo R., Shen-Orr S., Itzkovitz S., Kashtan N., Chklovskii D., and Alon U. Network motifs: simple building blocks of complex networks. *Science*, 298(5594):824–7, 2002. 7

[9] Borgatti S.P. Centrality and network flow. *Social Networks*, 27:55–71, 2005. 12

[Index]
